# Chondroitin sulphate *N*-acetylgalactosaminyl-transferase-1 inhibits recovery from neural injury

**DOI:** 10.1038/ncomms3740

**Published:** 2013-11-12

**Authors:** Kosei Takeuchi, Nozomu Yoshioka, Susumu Higa Onaga, Yumi Watanabe, Shinji Miyata, Yoshino Wada, Chika Kudo, Masayasu Okada, Kentaro Ohko, Kanako Oda, Toshiya Sato, Minesuke Yokoyama, Natsuki Matsushita, Masaya Nakamura, Hideyuki Okano, Kenji Sakimura, Hitoshi Kawano, Hiroshi Kitagawa, Michihiro Igarashi

**Affiliations:** 1 Department of Neurochemistry and Molecular Cell Biology, Brain Research Institute, Niigata University, 1-757 Asahi-machi, Niigata 951 8510, Japan; 2 Center for Transdisciplinary Research, Brain Research Institute, Niigata University, 1-757 Asahi-machi, Niigata 951 8510, Japan; 3 Laboratory of Neural Regeneration, Tokyo Metropolitan Institute of Medical Science, 2-1-6 Kamikitazawa, Tokyo 156 8506, Japan; 4 Doctoral and restart postdoctoral fellowship program, Japan Society for the Promotion of Science (JSPS), Tokyo 102 8472, Japan; 5 Department of Biochemistry, Kobe Pharmaceutical University, 4-19-1 Motoyamakita-machi, Kobe 658 8558, Japan; 6 Department of Neurosurgery, Brain Research Institute, Niigata University, 1-757 Asahi-machi, Niigata 951 8510, Japan; 7 Department of Dermatology, Graduate School of Medical and Dental Sciences, Brain Research Institute, Niigata University, 1-757 Asahi-machi, Niigata 951 8510, Japan; 8 Department of Comparative and Experimental Medicine, Brain Research Institute, Niigata University, 1-757 Asahi-machi, Niigata 951 8510, Japan; 9 Translational Research Center (TRC), Ehime University Hospital, Shitsukawa, Ehime 791-0295, Japan; 10 Department of Orthopedics, Keio University School of Medicine, 35 Shinanomachi, Tokyo 160 8582, Japan; 11 Departments of Physiology, Keio University School of Medicine, 35 Shinanomachi, Tokyo 160 8582, Japan; 12 Department of Cellular Neurobiology, Brain Research Institute, Niigata University, 1-757 Asahi-machi, Niigata 951 8510, Japan

## Abstract

Extracellular factors that inhibit axon growth and intrinsic factors that promote it affect neural regeneration. Therapies targeting any single gene have not yet simultaneously optimized both types of factors. Chondroitin sulphate (CS), a glycosaminoglycan, is the most abundant extracellular inhibitor of axon growth. Here we show that mice carrying a gene knockout for CS *N*-acetylgalactosaminyltransferase-1 (T1), a key enzyme in CS biosynthesis, recover more completely from spinal cord injury than wild-type mice and even chondroitinase ABC-treated mice. Notably, synthesis of heparan sulphate (HS), a glycosaminoglycan promoting axonal growth, is also upregulated in TI knockout mice because HS-synthesis enzymes are induced in the mutant neurons. Moreover, chondroitinase ABC treatment never induces HS upregulation. Taken together, our results indicate that regulation of a single gene, *T1*, mediates excellent recovery from spinal cord injury by optimizing counteracting effectors of axon regeneration—an extracellular inhibitor of CS and intrinsic promoters, namely, HS-synthesis enzymes.

## Abstract (web summary)

The glycosaminoglycan chondroitin sulphate inhibits axon growth. Here the authors show that mice deficient in chondroitin sulphate biosynthesis have increased levels of heparan sulphate, which is more efficient than chondroitinase in supporting recovery from spinal cord injury.

## Graphical abstract

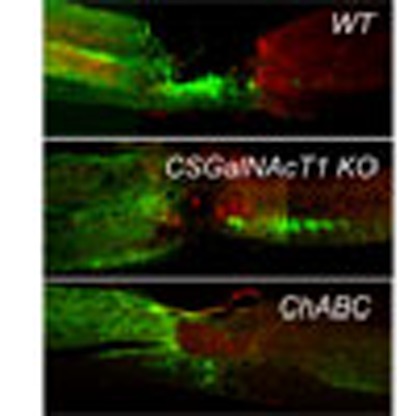

Many patients with spinal cord injury (SCI) suffer from severe paralysis^1^,^2^, possibly because injured axons in the adult mammalian central nervous system (CNS), including those in humans, rarely regenerate^1^,^2^. Recent findings demonstrate that the regulation of both extracellular and intrinsic factors that affect axon regeneration is essential to recovery from adult CNS neuronal injury^3^,^4^. Reduction or overexpression of intracellular cell-autonomous regulators promotes axon regrowth^5^,^6^,^7^,^8^,^9^,^10^,^11^; however, controlled manipulation of the endogenous expression of these molecules is challenging^8^. Regrettably, current protocols for removing or reducing the extracellular inhibitors do not result in axon regeneration sufficient for complete recovery from SCI.

Chondroitin sulphate (CS), a glycosaminoglycan (GAG) produced mainly by reactive astrocytes and partly by NG2 proteoglycan-positive cells after SCI, is the most widely distributed and most potent inhibitor of axon regeneration^3^,^5^,^6^,^7^,^12^. CS degradation resulting from application of chondroitinase ABC (ChABC), a bacterial enzyme, to the injury site promotes some additional axon regeneration^6^. Many practical methods can effectively provide ChABC near injury sites; notably, methods involving viral vector delivery of *ChABC*^13^,^14^,^15^ result in more axon sprouting^16^ (see reviews^17^,^18^). In addition, we must consider treatment alternatives; specifically, we must recognize that CS may be physiologically necessary to minimize the inflammation and limit its area after SCI by inhibiting invasion of macrophages^19^,^20^. Thus, complete removal of CS may exacerbate, not ameliorate, SCI.

Here we focused on optimizing CS synthesis following SCI. The process of the CS synthesis is very complicated, and more than 10 enzymes participate in CS synthesis^21^,^22^,^23^ (Fig. 1a). Notably, heparan sulphate (HS) is also a GAG and a potent promoter of axonal growth, and the first enzymatic step of CS synthesis and of HS synthesis are the same^24^. Therefore, the second step in CS synthesis, catalysed by CS *N*-acetylgalactosaminyltransferase-1 (abbreviated as T1 in this paper), is the first unique and rate-limiting step in CS synthesis; consequently, T1 is tightly regulated because it begins CS-specific polysaccharide chain synthesis^24^,^25^,^26^,^27^,^28^.

Here we generated T1-knockout (T1KO) mice^29^ and induced SCI in these and wild-type (WT) mice to investigate the mechanism of recovery from compression-induced (70 kdyne impact force) SCI. T1KO mice exhibited significantly better recovery from SCI based on locomotor behaviours and histological analysis than did untreated WT or ChABC-treated WT mice. Synthesis of HS, as also upregulated in T1KO mice because expression of HS-synthesis enzymes was induced. Notably, ChABC treatment never induced HS upregulation. Judging from our results and those from two previous reports, an HS-synthesizing enzyme Ext2 satisfies the definition of a potential intrinsic growth regulator in neurons^30^,^31^. Our results indicate that a single gene, that encodes T1, is a promising therapeutic target for SCI treatment.

## Results

### T1KO recover more rapidly than WT and ChABC-treated mice

**Figure 1:**
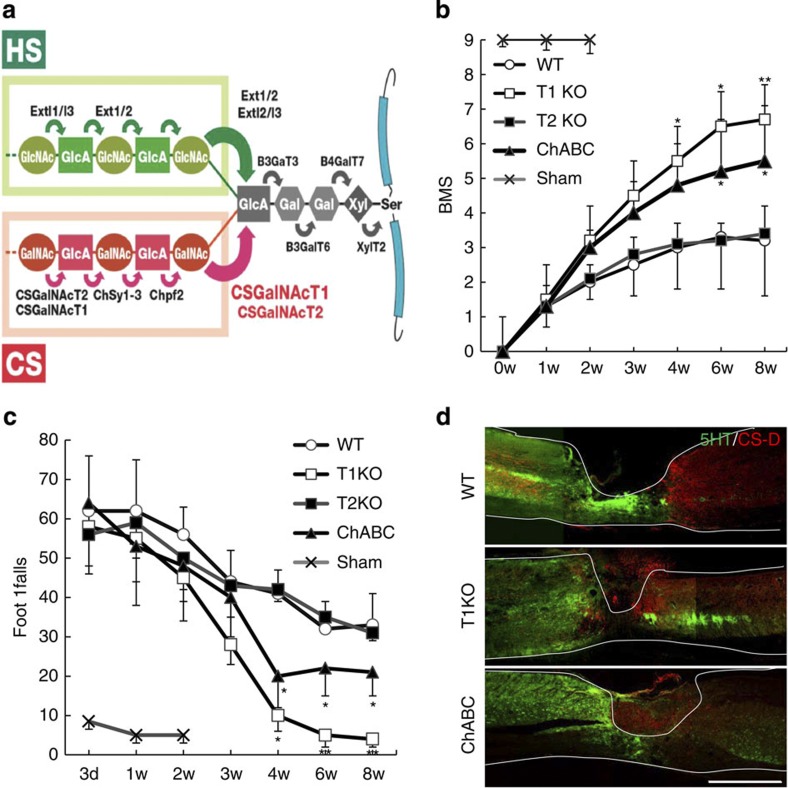
T1KO mice recover from SCI more quickly and more completely than WT mice. (**a**) Schematic diagram of GAG synthesis. The steps in CS synthesis are as follows: (1) synthesis of the tetrasaccharide linker that is attached to core proteins; (2) attachment of an *N*-acetylgalactosamine (GalNAc) to the linker; (3) addition of glucuronic acid (GlcA) to GalNAc and subsequent polymerization of the disaccharide backbone (GalNAc-GlcA); and (4) sulphation of several sites (**a**). During CS or HS synthesis T1 transfers GalNAc (CS) to the linker, whereas Ext1/Ext2 transfers GlcNAc (HS). T1 is primarily responsible for the catalysis of the first unique step in CS chain formation^21^,^22^,^26^,^28^ (**a**); therefore, T1 is the most important enzyme for regulation of CS synthesis. See Supplementary Table S2 for a description of enzyme abbreviations. Gal, galactose; GalNAc, *N*-acetylgalactosamine; GlcNAc, *N*-acetylglucosamine; GlcA, glucuronic acid; Xyl, xylose. (**b**) BMS scores after SCI. (**c**) Footfall tests after SCI. (**b**,**c**) T1KO versus WT and ChABC. *Post hoc* analyses were conducted using the Bonferroni–Dunn test for repeated-measures ANOVA. In **b** and **c**, data are expressed as the mean±s.e.m; *P<0.05; **P<0.01 (*n*=9; ANOVA). (**d**) Immunohistochemistry of 5HT(+) axon terminals in mice 8 weeks after SCI. 5HT (green) and CS (red). Scale bars, 1 mm.

We had focused on optimizing CS synthesis following SCI. However, the formation of a tetrasaccharide linker is notably the first step in both CS and HS biosynthesis, and CS inhibits axonal regrowth, whereas HS promotes it (Fig. 1a). Therefore, the second step in CS and HS synthesis—which is catalysed by T1 and the Ext1-Ext2 heterodimer (Ext1/Ex2), respectively—is the first unique and rate-limiting step in each pathway. These enzymes share a substrate, the tetrasaccharide linker^21^, and they bifurcate GAG synthesis into two alternative pathways^24^,^26^. Although CS *N*-acetylgalactosaminyltransferase-2 (CSGalNAcT2; abbreviated as T2 in this paper) is a T1 isoform, it has lower enzyme activity than does T1^21^,^27^.

We focused on reducing T1 activity, via gene knockout, as a potential treatment for SCI. T1KO mice are viable, but they have abnormal bone development and 10% shorter bodies than do WT mice^29^ (see Supplementary Fig. S1a–c). Following induced SCI, we examined and compared the recoveries of T1KO, T2-knockout (T2KO), untreated WT and ChABC-treated WT mice. Recovery of motor functions following SCI was evaluated using the Basso mouse scale (BMS) scoring^32^ (Fig. 1b), footfall tests (Fig. 1c; Supplementary Movie 1), footprint analysis (Supplementary Fig. S2a,b) and electromyography (Supplementary Fig. S2c); T1KO mice recovered from SCI more quickly and more completely than did T2KO, ChABC-treated WT or untreated WT mice (Fig. 1b,c; also see Supplementary Fig. S2d,e). The areas encompassing serotonin-positive (5HT(+)) terminals^1^ beyond the lesion site were much larger in T1KO than in untreated WT or ChABC-treated WT mice (Fig. 1d, Table 1 and Supplementary Fig. S2f,g), and there were many more 5HT(+) terminals in T1KO mice (Table 1; see also Supplementary Fig. S2f,g). These observations indicated that functional recovery after SCI was associated with axon regrowth and/or sprouting (Fig. 1d and Table 1) in T1KO mice. Importantly, these effects in T1KO mice were superior to those following ChABC treatment (Table 1 and Supplementary Fig. S2g), and T2KO mice did not show any significant increase in 5HT(+) staining (Fig. 1e and Supplementary Fig. S2f,g). To determine whether the corticospinal tract (CST; a representative pathway operating voluntary movement) recovered, we traced the biotinylated dextran amine (BDA) (+)-extending axons in each group of mice after SCI. Axons that had migrated past a scar (Supplementary Fig. S3a,b) and into caudal regions were evident only in T1KO SCI mice (Supplementary Fig. S3b). GAP-43 immunoreactivity—which is a marker of neuron growth, growth cones and extending axons^14^,^33^—was evident in CST axons in caudal regions only in T1KO SCI mice, but not in ChABC-treated WT or T2KO SCI mice (Supplementary Fig. S3c). Following SCI, caudal GAP-43(+)-CST axon terminals were significantly more abundant in T1KO SCI mice than in WT or T2KO mice (Supplementary Fig. S3d,e).

**Figure 2:**
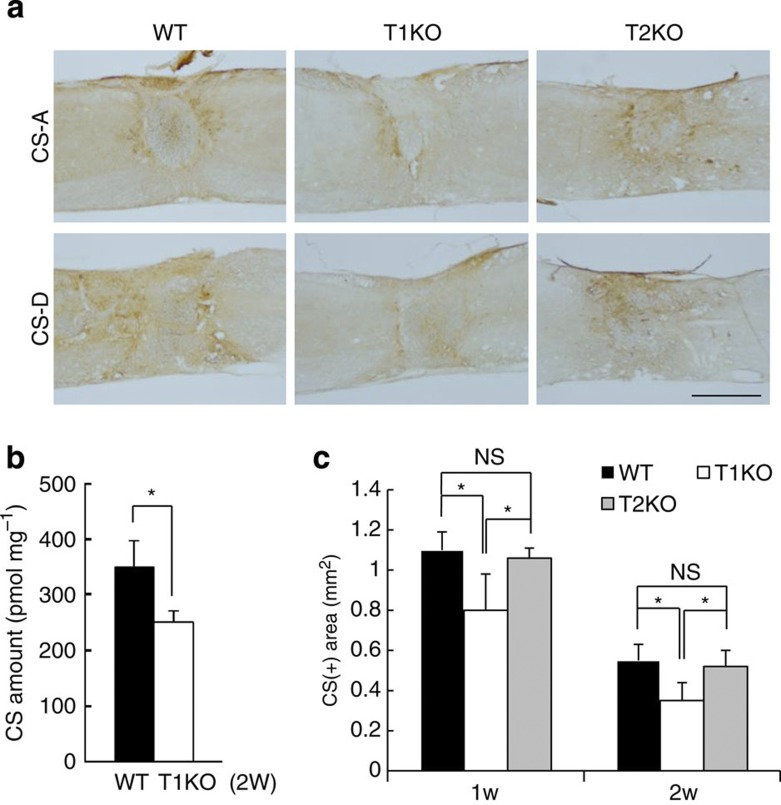
CS synthesis after the CSI is lower in T1KO than in WT mice. (**a**) Following SCI, CS expression 2 weeks after the injury was considerably lower in T1KO mice than in WT or T2KO mice. Anti-CS-A and anti-CS-D antibodies recognize two distinct sugar chain units of CS. Scale bar, 1 mm. (**b**) CS accumulation following SCI was lower in T1KO than in WT mice. *P<0.05 (Student’s *t-*test; *n*=5). (**c**) CS(+) areas were smaller in T1KO (grey) mice than in WT (white) or T2KO (grey) mice. Repeated-measures ANOVA followed by the Bonferroni–Dunn test. *P<0.05 (*n*=6). Data are expressed as the mean±s.e.m. in **b** and **c**.

**Figure 3:**
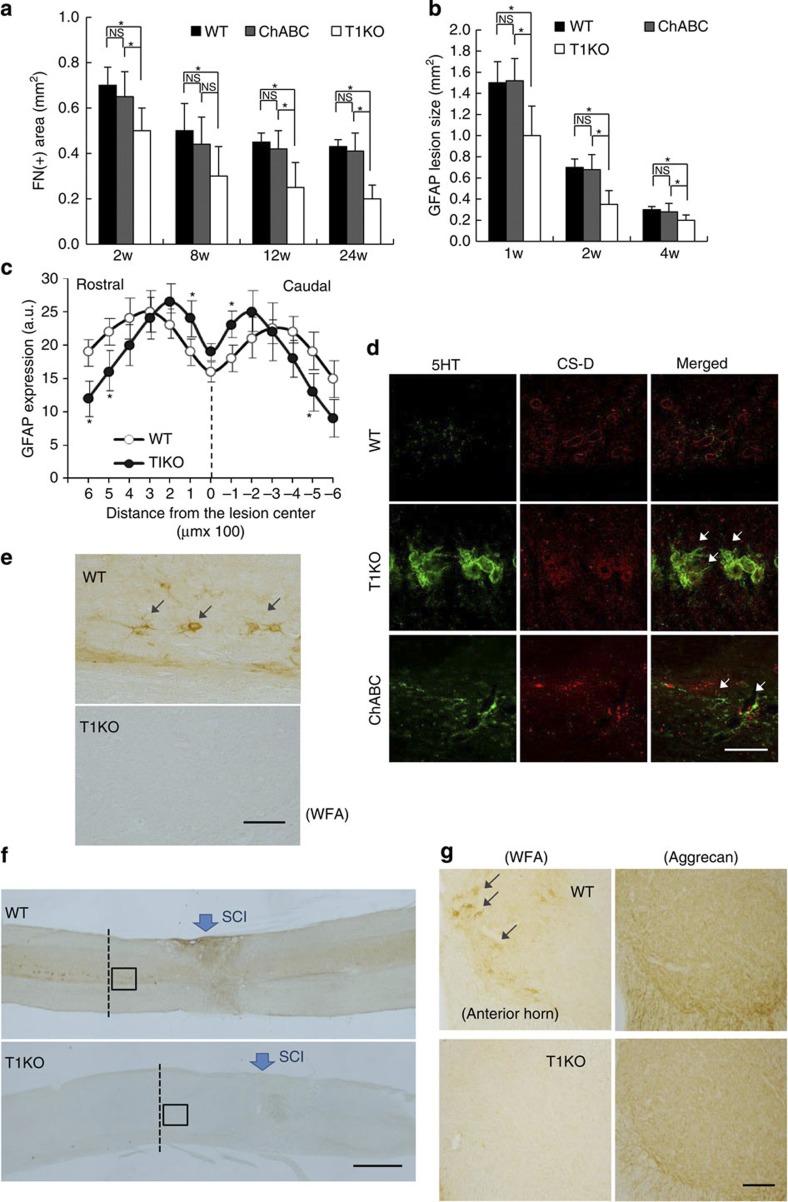
Reduced CS levels are associated with reduced scar formation in T1KO mice. In **a** and **b**, *n*=7; and in **c**, *n*=5. (**a**,**b**) Fibrotic scar areas (**a**) and glial scar areas (**b**) were smaller in T1KO mice (grey) than in WT (white) or in ChABC-treated mice (ChABC; black). Data are expressed as the mean±s.e.m. These data were compared by two-way ANOVA and Bonferroni’s *post hoc* pairwise comparisons; *P<0.05. (**c**) Glial scars in T1KO mice covered a narrow area that surrounded the lesion centre; these areas were narrower than those in WT mice (2 weeks after SCI). Data are expressed as the mean±s.e.m. Scheffe’s *post hoc* tests at each spinal segment showed significant differences between T1KO and WT mice at −5, −1 (caudal) and 1, 5, 6 (rostral) mm away from the lesion epicentre (*P<0.05). (**d**) Distribution of 5HT(+) terminals after recovery from SCI in T1KO mice was different from that in ChABC-treated (ChABC) mice. Many sprouting 5HT(+) terminals had accumulated in extracellular matrix around the cells in T1KO, but not in ChABC-treated mice. Arrows indicate the regrowing 5HT(+) terminals. Scale bar, 50 μm. (**e**–**g**) PNNs after SCI were not evident in T1KO mice. Higher (**e**) and lower (**f**) magnifications of WFA-labelled PNNs in WT and in T1KO 3 weeks after SCI. PNNs were evident in WT but not in T1KO mice. The boxed areas in **f** are magnified in **e**. Scale bars, 1 mm (**e**,**f**). (**g**) Coronal sections of the anterior horn were taken along the axis represented by the dotted line in **f** and stained via WFA or with anti-aggrecan antibody (aggrecan). In T1KO mice, WFA-labelled CS concentrated in PNN was not evident but signal from aggrecan, a core CSPG protein, was evident. Scale bars, 50 μm.

Next, we examined whether the superior recovery and axon regrowth in T1KO animals depended on reduction of CS. We used immunohistochemistry to confirm that ChABC treatment broke CS down (Supplementary Fig. S4a). T1 was highly expressed in some reactive astrocytes in WT mice following SCI, but it was not evident in T1KO mice (Supplementary Fig. S4b). Moreover, CS production in areas with glial scars, which are produced by reactive astrocytes^3^, was much lower in T1KO mice than WT or T2KO mice (Fig. 2a). We also demonstrated biochemically (Fig. 2b) and using morphometrical measurements (Fig. 2c) that CS levels in injured spinal cords of T1KO mice were lower than in those of WT or T2KO mice (Supplementary Fig. S4c). Undoubtedly, several other enzymes are involved in CS synthesis^28^ (*cf*. Fig. 1a), and CS is not completely absent from T1KO mice^29^ (Fig. 2c; Supplementary Fig. S4d). Notably, *Caenorhabditis elegans* lacks T1 and T2, yet a chondroitin backbone comprising GalNAc and GlcA is synthesized in worms by four other enzymes (ChSy1–3 and Chpf2) that are also found in mammals^34^ (see Fig. 1a); therefore, these four enzymes may synthesize CS in mammals lacking T1, although probably at a much slower rate than when T1 is present. Nevertheless, fibrotic scars (Fig. 3a) and glial scars^3^,^19^,^20^ (Fig. 3b) were much smaller in T1KO mice than in WT or ChABC-treated mice (Fig. 3a,b; Supplementary Fig S3a). In contrast, scar area did not differ significantly between ChABC-treated and WT mice (Fig. 3a,b). Each glial scar in T1KO mice was limited to a narrow area that surrounded the centre of the SCI lesion (Fig. 3c; Supplementary Fig. S3a). 5HT(+) terminals in T1KO mice were highly concentrated around the astrocytes but scars in ChABC-treated mice were not (Fig. 3d). These results indicated that both effects of T1KO—more complete axon regrowth and more regrowing axon terminals (Fig. 1b–d, and Table 1)—were related to a reduction in the ‘barrier’ scar area and that this reduction was a result of decreased CS production^3^,^6^,^7^ (Figs 2 and 3). Moreover, each effect was probably independent of residual CS in T1KO mice because ChABC treatment caused more CS degradation and led to worse outcomes than did T1KO (Fig. 3a,b and Table 1). ChABC treatment reportedly reduces the perineuronal net (PNN), which is enriched with CS and inhibits neural plasticity^35^; moreover, reductions in the PNN reportedly enhance neuronal plasticity, neuronal sprouting and recovery from SCI^36^,^37^. Therefore, we examined the PNN in mice recovering from induced SCI. We used *Wisteria floribunda* agglutinin (WFA), a generalized marker of PNN, to assess PNNs and found that WFA was evident in WT mice but not in T1KO mice (Fig. 3e–g).

### Increased HS synthesis in T1KO mice promotes rapid recovery

**Figure 4:**
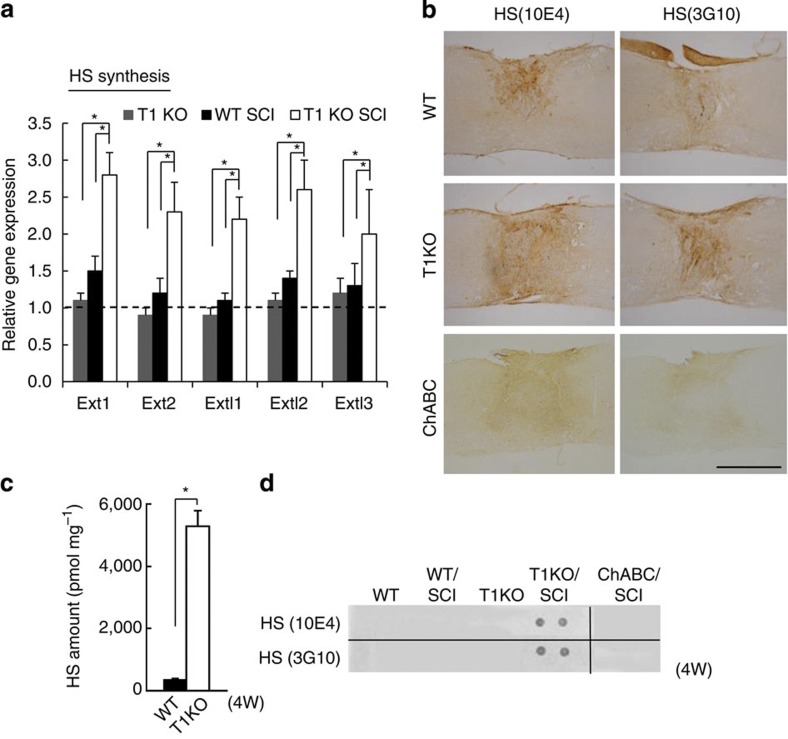
HS synthesis increases in injured spinal cords of T1KO mice. T1KO, T1-null; WT, wild type. Data are expressed as the mean±s.e.m. (**a,c**). (**a**) Expression of messenger RNAs encoding enzymes necessary for HS synthesis was assayed via reverse transcriptase–PCR. The level of mRNA expression in WT mice was defined as 1.0 for each gene. These data were compared by two-way ANOVA and Bonferroni’s *post hoc* pairwise comparisons. *P<0.05 (*n*=15); T1KO SCI versus WT SCI or versus T1KO (intact). (**b**) On the basis of immunohistochemistry, HS was highly expressed in T1KO mice but not in WT or ChABC-treated mice, 2 w after SCI. Antibodies 10E4 and 3G10 recognize different disaccharides in HS. Scale bar, 1 mm. (**c**) Biochemical quantification of HS expression at sites of SCI 2 weeks after injury. *P<0.05 (Student’s *t-*test; *n*=6). (**d**) Dot-blot analysis of HS expression in spinal cords before and after SCI (2 w). HS was detected only in T1KO mice with SCI, but not in any other mice, including the ChABC-treated mice.

On the basis of histological and phenotypic features of T1KO mice, we doubted that the 25% reduction in CS (Fig. 2c) in T1KO mice was solely responsible for such complete recovery from SCI (Fig. 1b–d, Table 1 and Supplementary Fig. S2a,b,d,e,g). Therefore, we suspected that changes in HS synthesis might also be involved because T1 and some HS-synthesis enzymes share the tetrasaccharide linker as a substrate^24^,^28^ (Fig. 1a). The expression of enzymes other than T1 that synthesize CS in response to SCI was not significantly different between injured T1KO and injured WT mice (Supplementary Fig. S5a). Surprisingly, however, the expression of HS-synthesis enzymes—including Ext1 and Ext2, which are essential to HS synthesis (Fig. 1a)—was much higher in injured T1KO mice than that in uninjured T1KO or injured WT mice (Fig. 4a). Importantly, ChABC treatment did not cause upregulation of these HS-synthesis enzymes (Supplementary Fig. S6a).

**Figure 5:**
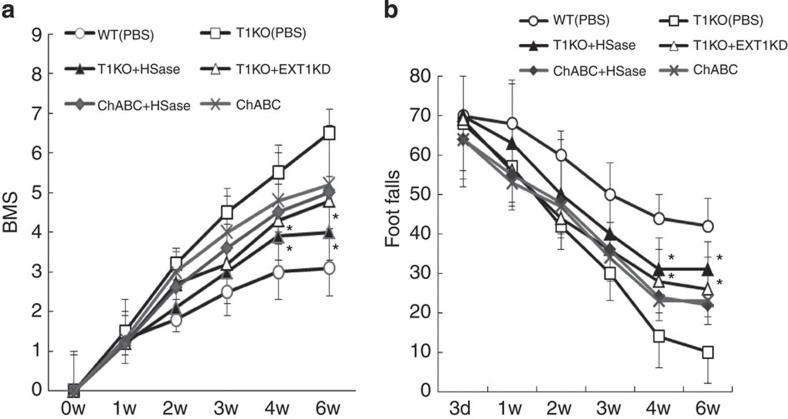
Increased HS promotes more rapid and more complete recovery from SCI. T1KO, T1-null; WT, wild type. Data are expressed as the mean±s.e.m. (**a**) BMS scores and (**b**) footfall tests with or without reduction of HS in WT, T1KO and ChABC-treated mice; ChABC was used at 0.2 U per 00 μl. HSase (0.4U per 200 μl; experimental treatment) or PBS (control treatment) was administered via a minipump. *EXT1KD*; KD of Ext1 by *in vivo* RNAi (100 nmol per 200 μl); also see Supplementary Fig. S8a. *Post hoc* analyses were conducted using the Bonferroni–Dunn test for repeated-measures ANOVA. *P<0.05 (*n*=5; in **a** and **b**). The BMS subscore for T1KO versus T1KO+HSase mice or versus T1KO+EXT1 KD were significantly different at 4 and 6 weeks after SCI.

On the basis of these results, we strongly suspected that HS synthesis contributed to the superior recovery of T1KO mice; therefore, we examined HS expression after SCI. HS-positive areas were significantly larger in the injured spinal cords of T1KO mice than in those of WT mice (Fig. 4b), and HS levels were 20-fold higher in the injured regions of T1KO mice than in those of WT (Fig. 4c; Supplementary Fig. S6b). **N**otably, neither ChABC treatment nor T2 knockout increased HS levels (Fig. 4b,d; Supplementary Fig. S6c). There were no significant differences between injured T1KO and injured WT mice in the expression of any CS-containing proteoglycan (CSPG) or syndecan-3, which is an HS-containing proteoglycan (HSPG; Supplementary Fig. S6a,d). To assess whether this upregulation of HS synthesis in T1KO mice following SCI promoted axon regrowth or sprouting, we examined the effects of continuously administered bacterial heparitinase (HSase), which degrades HS^30^, on recovery from SCI. On the basis of BMS scoring (Fig. 5a) and footfall tests (Fig. 5b), HSase treatment slowed the recovery of T1KO mice (Fig. 5 and Table 2), as did RNA interference (RNAi)-mediated knockdown (KD) of an HS-synthesis enzyme, Ext1 (Fig. 5 and Table 2). In addition, HSase treatment reduced the area encompassing regenerating 5HT(+) axon terminals in T1KO mice to a level similar to that in WT mice (Table 2), but the recovery in ChABC-treated mice was not sensitive to HSase, suggesting that ChABC did not induce HS upregulation (Supplementary Fig. S6a,e). These results indicated that upregulation of HS synthesis in T1KO mice promoted axon regrowth and/or sprouting and functional recovery from SCI, just as it promotes axon growth during CNS development^5^,^25^.

**Figure 6:**
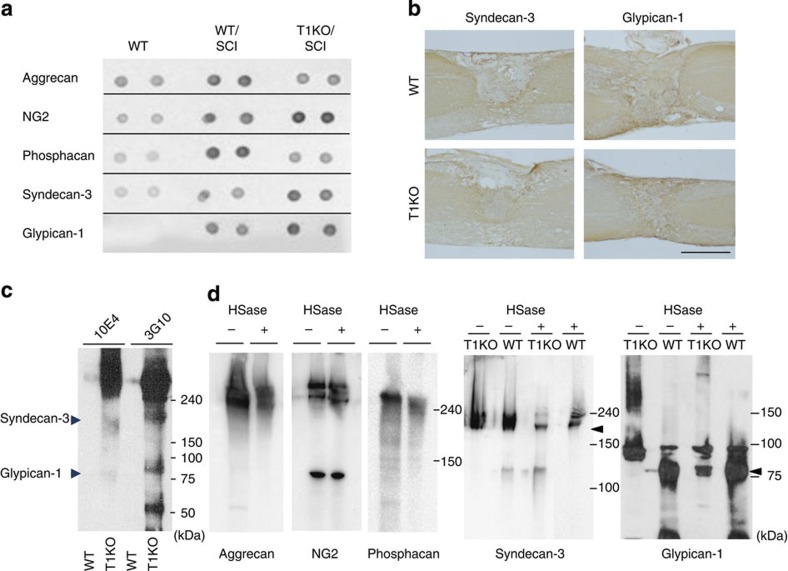
Syndecan-3 and glypican-1 are the target HSPGs of SCI-induced HS upregulation in T1KO mice. (**a**,**b**) Syndecan-3 and glypican-1, HSPGs derived from neurons, were expressed in T1KO mice after SCI (2 weeks). (**a**) Dot-blot analysis of the major core proteins decorated with CSPG or HSPG. (**b**) Immunohistochemistry of syndecan-3 and glypican-1 after SCI (2 weeks) in WT and in T1KO mice. Scale bar, 1 mm. (**c**) HSase-treated HSPGs detected by 3G10, the antibody that recognized the HS stub. At least three distinct HSPGs were recognized by 3G10 in T1KO SCI mice, but not in WT SCI mice, including 180 kDa (syndecan-3) and 65–80 kDa (glypican-1). (**d**) Western-blot analysis of PGs near the lesion sites before (***−***) and after (+) HSase treatment (5 mU; 37 °C, 3 h). Notably, syndecan-3 and glypican-1 were the main PGs with HSase-sensitive HS in injured T1KO mice. The data for CSPG core proteins (aggrecan, NG2 or phosphacan) are shown only for the injured T1KO mice, and each CSPG was HSase insensitive. Arrows indicate the position of deglycosylated proteins. Numbers on the right indicate molecular masses (kDa).

We tried to identify the target HSPGs in injured T1KO mice that bore the upregulated HSase-sensitive HS. HS-modified syndecan-3 (*N*-syndecan) and glypican-1 were highly expressed (Fig. 6a and Supplementary Fig. S6d) and widely distributed (Fig. 6b) at sites of SCI in T1KO mice. In the injured spinal cords of T1KO mice, both HSPGs were detected via HS-specific antibodies (Fig. 6c) and had HSase-sensitive HS chains (Fig. 6d); importantly, in WT SCI mice, degraded HS derived from these two HSPGs was barely evident, indicating that the upregulated HSase-sensitive HS was specifically bound to syndecan-3 and glypican-1 in injured T1KO (Fig. 6d).

**Figure 7:**
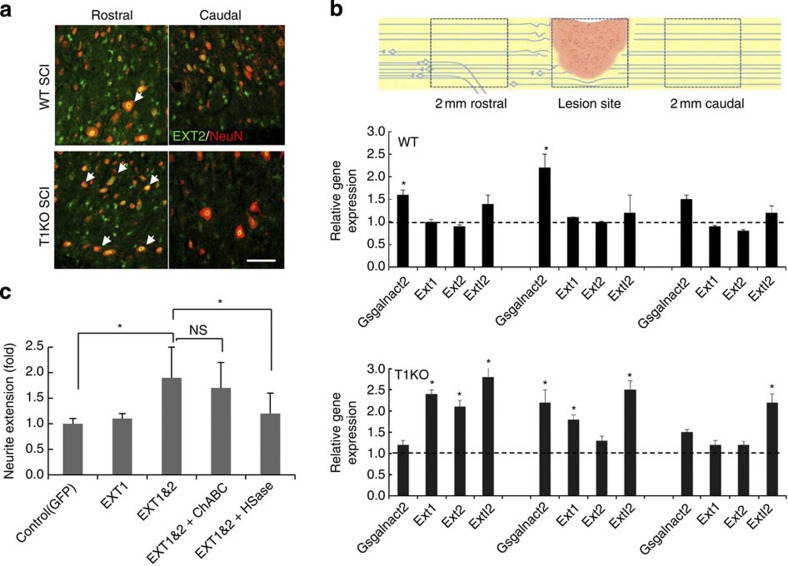
Elevated expression of HS is necessary to axon regrowth. WT/SCI, WT after SCI; KO/SCI, T1KO after SCI. (**a**) Ext2 (green), an essential enzyme for HS sugar chain synthesis, was upregulated in and localized to neurons of T1KO mice after SCI. NeuN (red), a neuronal marker; Ext2-expressing cells (arrows). Scale bars, 20 μm. (**b**) Reverse transcriptase (RT)–PCR of genes encoding enzymes involved in GAG synthesis after SCI. The samples were collected from three sites in wild-type (WT) or T1KO mice: 2 mm rostral from the lesion centre (left), the lesion centre (centre) and 2 mm caudal from the lesion centre (right). The samples were analysed using RT–PCR. Notably, the rostral site was mainly composed of neurons, not of reactive astrocytes (the left-most cartoon). The average expression of each gene in intact WT or in intact T1KO mice was defined as 1.0. Expression of each gene (*Csgalnact2 (T2), Ext1, Ext2* or *Extl2*) in TIKO versus that in *WT*. *P<0.05 (*n*=6; Bonferroni’s comparison test). (**c**) Overexpression of Ext1/Ext2 promoted axon outgrowth of WT cortical neurons in an HSase-dependent manner. Both *Ext1* and *Ext2* complementary DNAs (Ext1 and 2) were transfected into cultured neurons. The Ext1 and Ext2 proteins are thought to form a heterodimeric complex during HS synthesis; overexpression of *Ext1* alone did not result in a significant change. *P<0.05 (one-way ANOVA; *n*=4); *Ext1&2* versus control (*GFP*) or versus *Ext1&2+*HSase. In each individual experiment, 100 neurons were counted and the experiments were performed at least three times. Data are expressed as the mean±s.e.m. in **b** and **c**.

After SCI, Ext2 was expressed in neurons^30^ of T1KO mice (Fig. 7a), and the expression of HS-synthesis enzymes was upregulated in neuron-rich areas rather than in areas of glial scarring (Fig. 7b); therefore, we concluded that extra HS was produced by the neurons^5^,^24^,^25^ that expressed HS-synthesis enzymes such as Ext2. Receptor protein tyrosine phosphatase-σ (RPTPσ), a receptor for both HSPGs and CSPGs^38^,^39^, was also upregulated in T1KO mice after SCI, and RPTPσ co-localized with some of the regrowing and/or sprouting axons (Supplementary Fig. S7a–d). HS, unlike CS, is known to promote axon growth during neural development^5^,^24^,^25^,^31^,^39^; therefore, our data indicated that GAG synthesis in T1KO mice was more favourable to axon regrowth than was the GAG synthesis in WT mice. To assess whether upregulation of HS was directly involved in neurite growth following SCI and whether effects of RPTPσ on neurite growth depended on HS, we examined the effects of concurrent overexpression of Ext1 and Ext2 on cultured neurons (Fig. 7c). Ext1-Ext2 overexpression induced neurite growth in an HSase-sensitive (Fig. 7c) and RPTPσ-sensitive (Supplementary Fig. S7e) manner^39^, indicating that upregulation of HS contributed to axon growth. Therefore, HS-synthesis enzymes such as Ext2 may promote axon growth intrinsically in neurons by targeting new HS synthesis to syndecan-3, glypican-1 or both^40^. *RPTPσ* messenger RNA expression and RPTPσ levels were elevated in WT, ChABC-treated and T1KO mice (Supplementary Fig. S7c,d), indicating that at least the increased axon regrowth was not due to a RPTPσ-dependent reduction in CS.

RNAi-mediated T1 KD (T1-KD) *in vivo* led to excellent recovery from SCI (Supplementary Fig. S8a–e). T1-KD-associated scars were similar in size to T1KO-associated scars (Supplementary Fig. S8f). T1-KD caused reduced CS synthesis (Supplementary Fig. S8g), and scar sizes were significantly smaller following T1-KD than following ChABC treatment (Supplementary Fig. S8h). Moreover, downregulation of CS synthesis and upregulation of HS-synthesis enzymes were induced by simultaneous T1-KD and T2-KD, as well as by T1KO (Supplementary Fig. S9a–c and Fig. 4, also see Supplementary Fig. S5a,b); notably, T1-KD alone was insufficient to cause both effects (Supplementary Fig. S9c,d; compared with Supplementary Fig. S9a,b).

## Discussion

**Figure 8:**
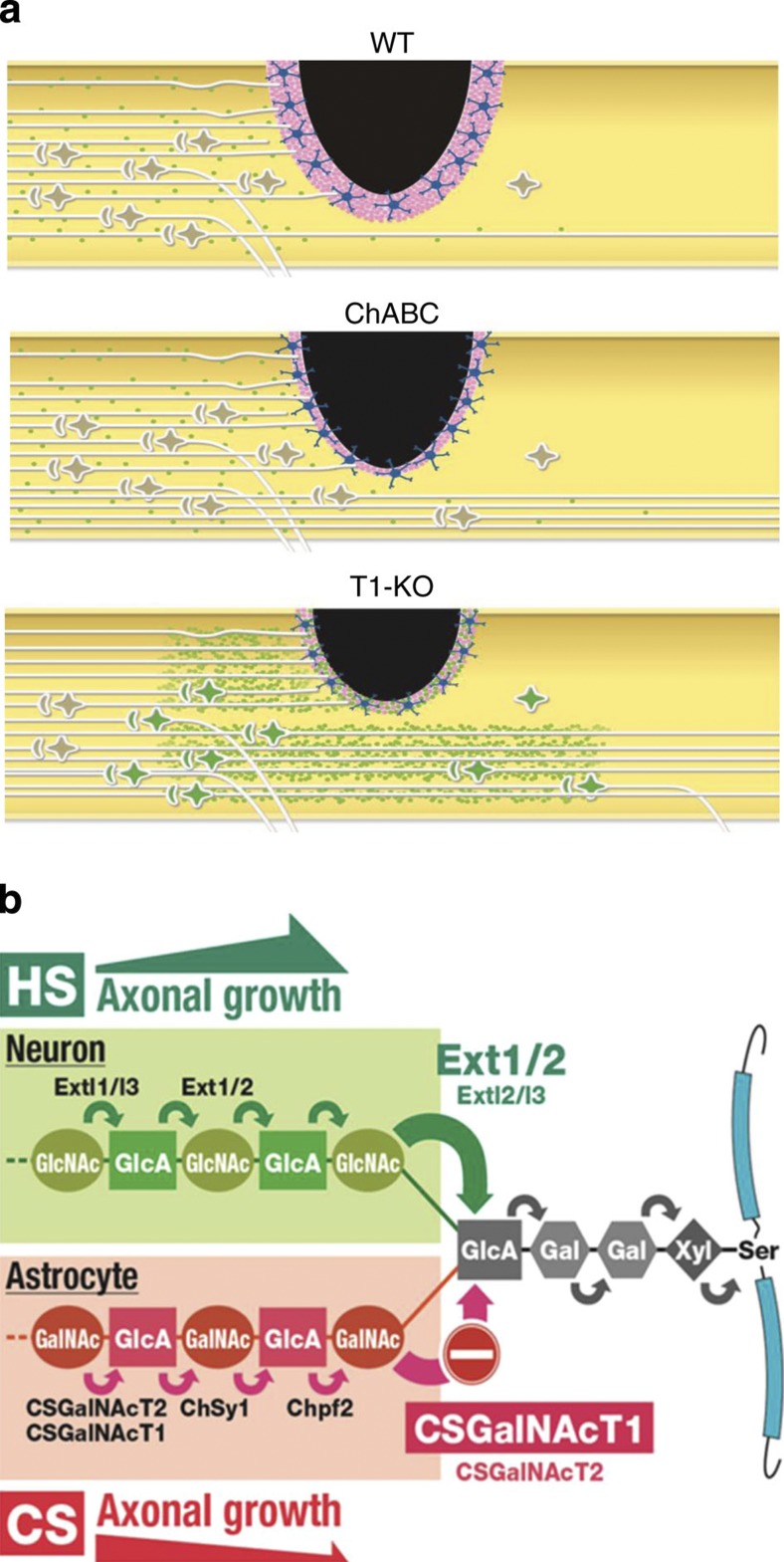
Schematic model of T1-regulated GAG balance after SCI. (**a**) Summary of the results on SCI recovery. In WT (uppermost) mice, CS (dark pink) production was elevated in the reactive astrocytes (star-like form in blue), and thick scars (black)^3^, which inhibited axon regeneration, formed; consequently, very few regenerating axons could migrate to areas past the SCI lesion. In contrast, in T1KO mice (middle), CS production was reduced and the scars were smaller than those in WT mice. In addition, HS (green) expression was high in T1KO neurons, and the number of regrowing or sprouting axons was higher than that in WT. Although ChABC-treated mice (ChABC; lowermost) had less CS than did WT or T1KO, they had larger scars than T1KO mice, and they did not overproduce HS; therefore, axonal regeneration was reduced and restricted relative to that in T1KO. WT and ChABC-treated mice, unlike T1KO mice, experienced only baseline levels of HS synthesis after SCI; these baseline levels could not promote recovery from SCI. (**b**) Possible biochemical mechanism that promotes better recovery from SCI in TIKO mice^26^. In WT, T1 is upregulated in reactive astrocytes after SCI; however, *Ext1* and *Ext2*, which are genes with putative ‘axon growth-promoting activity/potential’, are upregulated in neurons of T1KO mice, and T1 is not expressed (minus in red); consequently, HS, rather than CS, accumulates around T1KO neurons. As a result, the amount of CS decreases and that of HS increases, and this shift in the CS-HS balance promotes axon regrowth^39^. As increases in HS and decreases in CS persist, the potential for axon growth is elevated.

T1KO mice had significantly better recovery from SCI than did WT or ChABC-treated mice (Fig. 1b–d); three distinct phenomena were responsible for this superior recovery. First, T1KO reduced CS synthesis and resulted in smaller scars than did ChABC treatment; reduced scaring probably contributed to better recovery because the smaller scars introduced smaller physical and weaker chemical barriers to the regrowing or the sprouting axons (Figs 1e, 2c and 3a–d, and Supplementary Fig. S2f). In addition, T1KO probably reduced CS concentrations (Fig. 3e). Notably, reduction of CS was apparently superior to complete elimination of CS for SCI recovery, probably because CS has some positive roles in recovery (Fig. 8a). Second, simultaneous upregulation of HS synthesis and downregulation of CS in T1KO mice were essential to excellent recovery from SCI. HS promotes axon growth^5^,^24^,^25^, and syndecan-3, a target HSPG of SCI-induced HS upregulation, is enriched in axons and the extracellular matrix around axons^41^. T1KO and T1-KD+T2-KD (Supplementary Fig. S9c) produced the most favourable environment for recovery from SCI; specifically, these treatments shifted conditions at the injury away from the non-permissive CS-rich state (Fig. 8a,b). It is evident that T1KO was substantially superior to ChABC treatment specifically because HS synthesis was stimulated by T1KO and never by ChABC treatment (Fig. 5a,b, Table 2 and Supplementary Fig. S5a,d). HSPGs are important for signal transduction, axon growth and axon guidance^24^,^42^; therefore, our findings are very important to axon regeneration. Third, expression of HS chains on both syndecan-3 and glypican-1 and expression of HS-synthesis enzymes (for example, Ext2, Ext1 and Extl2) in neurons were essential to the superior recovery from SCI of T1KO mice (Figs 4a,d, 6a,c and 7a,b). Taken together, our results and those from two previous reports indicated that Ext2 satisfies the definition of a potential intrinsic growth regulator in neurons; the most relevant findings are that Ext1/2 expression is developmentally downregulated in the CNS^43^ and that Ext2 and syndecan-1 expressions in neurons are specifically upregulated during peripheral nerve regeneration^30^. Lack of T1 evidently induced these intrinsic positive regulators of axon growth^4^,^10^. These HS-synthesis enzymes belong to a newly identified group of ‘development-dependent factors with axon growth-promoting features’ and are totally different from previously described factors such as PTEN and KLFs^9^,^11^. Therefore, we conclude that T1KO and ChABC treatment led to recovery from SCI by very distinct mechanisms.

The following two strategies for controlling CS synthesis have been developed: first, a deoxyribozyme targeting *Xylt1* (see Fig. 1a) messenger RNA inhibits synthesis of the first residue of the tetrasaccharide linker^44^,^45^. Second, genetic ablation of Sox9, a transcription factor, inhibits expression of some CS-synthesizing enzymes (for example, Xylt1/2 and C4st1)^46^. Each strategy is effective in inhibiting CS synthesis and in improving recovery from SCI. However, based on our new findings, inhibition of T1 should be better than either of those strategies because both of those strategies should inhibit HS synthesis, and HS synthesis promotes axon growth.

In conclusion, our results demonstrate that knocking out a single enzyme, T1, caused both reduced CS synthesis and increased HS synthesis and that manipulation of this single enzyme resulted in the reduction of extracellular inhibitors and the induction of intrinsic growth promoters of axon regeneration. Therefore, our findings provide a novel principle for development of axon regeneration strategies. Chemicals that specifically inhibit T1 might be effective treatments for SCI; however, we need to know the molecular mechanisms by which neuronal HS-synthesis genes (for example, *Ext2*) were upregulated in T1KO mice. Finally, adding inhibition of TI to other treatments^36^,^47^ for SCI may improve overall clinical outcomes.

## Methods

### Materials

ChABC and HSase (HSase I/III), heparanase I and II, WFA and AteloGene Local Use Kits for atelocollagen-mediated *in vivo* short interfering RNA were purchased from Seikagaku Corp. (Tokyo, Japan), from IBEX Technology Inc. (Montreal, Canada), Vector Laboratories Inc. (Burlingame, CA, USA) and from KOKEN Co. Ltd. (Tokyo, Japan), respectively. An osmotic minipump (Model 2006 (for 7W long) and Model 2004 (for 4W long)), catheter tubing (Mit-02) and the infusion kit were purchased from ALZET (Cupertino, CA). Antibodies used in this study are listed in Supplementary Table S1 (As for the two antibodies provided from other researchers, listed in this Table, please refer to refs 48, 49).

### Generation of T2KO

All animal experimental studies were conducted with the approval of the Animal Care and Use Committee of Niigata University. T1KO^29^ mice were derived from the C57BL/6N strain. For most experiments, the WT strain used was C57BL/6N; the ICR strain was used only for the *in vivo* RNAi studies. T2KO mice were generated via the methods used to generate T1KO mice^31^. The mouse *T2* gene was identified as NM_030165. Exon 5 of mouse *T2* encodes a DXD motif, which is a binding site for Mn^2+^ that is essential to the *in vitro* activity of most GalNAc/Gal transferases^50^; therefore, we designed a targeting vector with the mouse *T2* exon 5 located between two loxP sites (Supplementary Fig. S1a). The T2KO mice were generated using an embryonic stem (ES) cell line derived from C57BL/6N mice; this line is designated RENKA. Resulting chimeric mice were mated to C57BL/6N mice and heterozygous offspring (*CSGalNAcT2*^*+/flox(neo)*^) were mated to *telencephalin-cre* mice^29^.

### Induction of SCI

Mice (8–10 weeks old, C57BL/6J, male) were subject to compression-induced SCI; a bilateral contusion injury was induced at the 10th thoracic vertebrae (Th10) of individual mice with a commercially available SCI device (Infinite Horizon Impactor; Precision Systems and Instrumentation, Lexington, NY)^20^,^32^, except that here we used a 70-kdyne impact force. The device reports data regarding time versus force and time versus displacement. The locomotor recovery assessment was performed using video recording^32^, and BMS open-field scoring was used to test mice once weekly for functional recovery during the 6–8 weeks following SCI^32^. The evaluations were scored independently by two investigators who were unaware of the experimental groups (at least eight mice in each group). On the third day after induction of SCI, mice were excluded if they had an incomplete injury (BMS score>0 on that day). Morphometric assays were performed using SCI samples prepared from the sagittal sections at 12 μm intervals^32^.

For each footfall test, a mouse was placed on a wire mesh grid and videotaped for 5 min while on the grid. To be scored, an animal had to walk for a minimum of 3 of the 5 min as determined by three independent examiners; for each animal scored, the total number of the footfalls from the bars was counted, and the total walking time was recorded.

To assess stepping patterns of hind limbs during forward locomotion after injury, the bottom of a well-lit runway (3 cm wide and 100 cm long) was lined with white paper, and mice were required to run along the runway in a darkened box. To record footprints, the planter surfaces of hind limbs were brushed with ink during continuous locomotion across a paper.

The electrophysiological experiments to evaluate motor function resulting from evoked potential of the neuromuscular function were performed by short trains of five square-wave stimuli of 0.5 ms duration with an interstimulus interval of 3 ms (ref. 32). The active and the reference electrodes were placed in the muscle belly, and near the distal tendon of the muscle in each limb^32^, respectively. Scorps3 software (AD Instruments, New South Wales, Australia) was used to average and analyse 100 responses including the latency period.

### Quantitative real-time PCR

Total RNA was extracted from the SCI regions using an RNeasy FFPE kit or an ALL prep RNA kit (QIAGEN). Total RNA (0.5 μg) was used as template; the iScript one-step RT-PCR kit (Bio-Rad), the SsoFast Probes supermix (Bio-Rad) and the PrimeTime qPCR assay (Integrated DNA Technologies, IW) were use for real-time reverse transcriptase–PCR; the reaction parameters were as follows: 95 °C for 30 s; and 40 cycles at 95 °C for 2 s and 65 °C for 5 s; 65–95 °C for 2 s) as indicated in the Taqman probe (Life Technologies) gene expression assay materials. Nucleotide sequence of real-time quantitative PCR primers and ZEN double-quenched probes are listed in Supplementary Table S2.

### *In vivo* RNAi after SCI

For the *in vivo* RNAi studies, SCI was induced in ICR mice (8–10 w; Clea Japan, Inc.) with the IH impactor as described above except that the impact force was 100 kdynes. Just after the SCI contusion, gelfoam impregnated with a mixture containing one or two siRNAs (10 μM each) and AteloGene Local Use Kit (see Materials) was placed on the lesion area^51^,^52^,^53^. RNA sequences of the siRNAs used in these experiments are listed in Supplementary Table S3^54^. The scrambled sequences of the negative-control siRNAs had four or five nucleotides that differed from the corresponding nucleotides in the targeted siRNAs.

### ChABC and HSase treatments *in vivo*

Continuous ChABC treatment after SCI was performed using an osmotic pump^14^,^55^ set at the 10th thoracic vertebrae levels for up to 2 weeks (200 U ml^−1^ in a volume of 0.2 ml). For the HSase application, an osmotic minipump (ALZET, Cupertino, CA; Model 2006) was implanted subcutaneously and used to infuse HSase (0.2 unit per 6 μg) dissolved in saline containing 0.05% BSA or vehicle into the contusion site through a flexible plastic cannula because HSase is less stable than ChABC *in vivo*.

### Immunohistochemistry

The dilution ratios for each antibody are listed in Supplementary Table S1. Tissue sections were incubated with biotinylated secondary antibody and then with avidin–biotin peroxide complex^55^,^56^ (Vectastain ABC kit; Vector Laboratories Inc.). Digital images were taken with a confocal laser scanning microscope (Zeiss, LSM5 exciter) and AxioVision charge-coupled device camera (Carl Zeiss). Spinal cord cryosections (20 μm thick) were obtained from mice that had been transcardially perfused with 4% formaldehyde. Standardized areas for sampling^32^ in the central regions (10 sections) were calculated using Image J (NIH) and MetaMorph (Molecular Devices). For analysis of 5HT(+) sprouting axons and boutons, rectangular areas were set 1–3 mm distal to the central line of the lesion site in each section^32^, and 10 sagittal sections from each mouse were used for quantitative analysis.

### Anterograde labelling of the CST

Exactly 8 weeks after injury, BDA (10% in saline, molecular weight 10,000 Da; Molecular probes)^6^ was injected into the motor cortices to label the descending CST fibres. The injection site was precisely 2.1 mm posterior to the bregma, 2 mm lateral to the bregma and 0.7 mm deep. We performed pressurized injections with a glass capillary attached to a microsyringe (Narishige) at a rate of 0.1 μl min^−1^ until the desired amount was injected. Exactly 2 weeks after each injection, the animals were anaesthetized and perfused with PBS and then with 4% paraformaldehyde in PBS. We used Alexa Fluor 488-conjugated streptavidin (Invitrogen) and the Vectastain ABC Elite kit (Vector Laboratories Inc.) or a tyramide signal amplification fluorescence system (Perkin Elmer) to fluorescently label the BDA.

### Quantification analysis of GAGs from the spinal cord

GAG analyses were conducted by enzymatic treatment and HPLC-based quantification^29^,^57^. GAGs were extracted from spinal cord tissue samples by incubating the samples in a protease solution (0.01 mg actinase E, 10 mM CaCl_2_, 50 mM Tris–HCl (pH 8.0)) at 55 °C for 2 days. After addition of trichloroacetate, each extract was centrifuged at 15,000 *g* for 20 min. Each partially purified CSPG and HSPG fractions was digested with ChABC (5 mIU ChABC in 60 mM CH_3_COONa, 50 mM Tris–HCl (pH 8.0)) or HSase (0.5 mIU HSase in 20 mM CH_3_COONa, 2 mM (CH_3_COO)_2_Ca (pH 7.0)), respectively. GAGs in each digests were derivatized using 2-aminobenzamide; these mixtures were analysed using HPLC (column: YMC pack PA, elution: 16–530 mM NaH_2_PO_4_)^57^.

### Neuronal cell culture

Cortical neurons were prepared from embryonic day 17 or postnatal day 1 mice^33^, and were then transfected with a human Ext1 construct in a pCMVvector (Takara Bio Co., Otsu, Japan), a human Ext2 construct in the pcDNA3.1 vector (Invitrogen) or a combination thereof. Stealth Select RNAi (Invitrogen, s72517, s72516 and s72518) was used to knockdown RPTPσ expression. In some cultures, ChABC (2 mU) or HSase (2 mU) was added 12 h after transfection. Cells were stained with anti-HS 10E4 antibody 48 h after transfection. The sequences of siRNAs targeting RPTPσ are listed in Supplementary Table S3. siRNAs with scrambled sequence (Invitrogen) were used as negative controls.

### Statistics

Data are presented as mean values±s.e.m. Prism version 5.04 software (GraphPad) or SPSS statistics software (IBM) were used for to perform statistical analyses. Unpaired two-tailed Student’s *t-*tests were used for single comparisons. Analysis of variance (ANOVA; one-way, two-way or repeated-measures ANOVA) were used for multiple comparisons; Bonferroni’s procedure were used to assess *post hoc* pairwise differences. Repeated-measures ANOVA was used to analyse BMS and footfall scores with follow-up comparison of treatments for each day, by contrast *t*-test and correction for multiple comparisons by the Holm method (*P*=0.05). Each investigator was blinded with regard to genotypes during all procedures performed to collect data for subsequent statistical analysis. For analyses of the morphometric data, all evaluations were performed by ANOVA with *post hoc*-independent pairwise analysis. *P*<0.05 was considered statistically significant. The specific tests used to analyse data from each set of experiments are indicated in the figure legends.

## Author contributions

M.I. and K.T. designed the experiments, and analysed the data. Y. Watanabe, K.Oh, K.Od, T.S., M.Y. and K.S. produced the gene-targeted mice. N.Y., S.H.O., M.O., C.K., Y. Wada and H. Kawano developed the SCI model and performed the biochemical and the histological experiments. N.M. designed the siRNA sequences. M.N. and H.O. taught members of the lab the techniques necessary for the SCI model. S.M. and H. Kitagawa performed the quantitative analysis of CS and HS. M.I. wrote the paper, and in part, K.T. wrote the Methods section.

## Additional information

**How to cite this article:** Takeuchi, K. *et al.* Chondroitin sulphate *N*-acetylgalactosaminyltransferase-1 inhibits recovery from neural injury. *Nat. Commun.* 4:2740 doi:10.1038/ncomms3740 (2013).

## Supplementary Material

Supplementary Figures and Tables Supplementary Figures S1-S9 and Supplementary Tables S1-S3

Supplementary Movie 1 Video of motor function recovery after SCI. WT and T1KO mice 4 weeks after SCI. The mice were placed on a steel net and the recovery of the walk was recorded. Note that the T1KO mouse walked much smoother than the WT mouse.

## Figures and Tables

**Table 1:** Quantification of areas encompassing 5HT(+) terminals.

	**2**~**3 mm rostral**	**Lesion**	**2~3 mm caudal**	**4**~**5 mm caudal**
WT	8,780±1,345	926±21	85±10	30±11
T1KO	8,942±1,137	365±32	2,779±897*	2,025±910*
T2KO	8,765±1,029	902±41	72±12	26±13
ChABC	6,238±3,547	643±92	779±615*	1,151±505*

ChABC, ChABC-treated; Sham, Sham-operated.

The areas encompassing 5HT(+) terminals in the ventral horn were measured 6 weeks after injury^32^ (see also Supplementary Fig. S2e). Data are expressed as the mean ±s.e.m. ANOVA and Bonferroni’s multiple comparison test (Prism 5.04); *n*=9; *P<0.05 (versus WT).

**Table 2:** Quantitative analysis of the area of the caudal region covered by 5HT(+) terminals after HSase treatment 4 weeks after injury.

	**2**~**3 mm rostral**	**Lesion**	**2~3 mm caudal**	**4**~**5 mm caudal**
WT+PBS	8,501±1,522	799±31	81±11	32±14
T1KO+PBS	8,862±1,204	415±51	2,398±991*	2,012±813*
T1KO+HSase	5,703±3,134	512±107	719±337*	698±173*
ChABC+HSase	6,021±3,467	740±153	800±593*	1,003±566*

ChABC, ChABC-treated; HSase, HSase-treated.

These data were compared by two-way ANOVA and Bonferroni’s multiple comparison test (Prism 5.04). T1KO+PBS versus T1KO+HSase, WT+PBS or ChABC+HSase.

*P<0.05 (*n*=5)

## References

[b1] Tuszynski M. H. & Steward O. Concepts and methods for the study of axonal regeneration in the CNS. Neuron 74, 777–791 (2012). 22681683 10.1016/j.neuron.2012.05.006 PMC3387806

[b2] Esposito E. & Cuzzocrea S. Anti-TNF therapy in the injured spinal cord. Trends Pharmacol. Sci. 32, 107–115 (2011). 21185611 10.1016/j.tips.2010.11.009

[b3] Silver J. & Miller J. H. Regeneration beyond the glial scar. Nat. Rev. Neurosci. 5, 146–156 (2004). 14735117 10.1038/nrn1326

[b4] Liu K., Tedeschi A., Park K. & He Z. Neuronal intrinsic mechanisms of axon regeneration. Annu. Rev. Neurosci. 34, 131–152 (2011). 21438684 10.1146/annurev-neuro-061010-113723

[b5] Bandtlow C. E. & Zimmermann D. R. Proteoglycan in the developing brain: new conceptual insights for old proteins. Physiol. Rev. 80, 1267–1290 (2000). 11015614 10.1152/physrev.2000.80.4.1267

[b6] Bradbury E. J. *et al.* Chondroitinase ABC promotes functional recovery after spinal cord injury. Nature 416, 636–640 (2002). 11948352 10.1038/416636a

[b7] Carulli D., Laabs T., Geller H. M. & Fawcett J. W. Chondroitin sulfate proteoglycans in neural development and regeneration. Curr. Opin. Neurobiol. 15, 116–120 (2005). 15721753 10.1016/j.conb.2005.01.014

[b8] Lee J. K. *et al.* Combined genetic attenuation of myelin and Semaphorin-mediated growth inhibition is insufficient to promote serotonergic axon regeneration. J. Neurosci. 30, 10899–10904 (2010). 20702718 10.1523/JNEUROSCI.2269-10.2010 PMC2974627

[b9] Liu K. *et al.* PTEN deletion enhances the regenerative ability of adult corticospinal neurons. Nat. Neurosci. 13, 1075–1083 (2010). 20694004 10.1038/nn.2603 PMC2928871

[b10] Yang P. & Yang Z. Enhancing intrinsic growth capacity promotes adult CNS regeneration. J. Neurol. Sci. 312, 1–6 (2012). 21924742 10.1016/j.jns.2011.08.037

[b11] Moore D. L. *et al.* KLF family members regulate intrinsic axon regeneration ability. Science 326, 298–301 (2009). 19815778 10.1126/science.1175737 PMC2882032

[b12] Jones L. L., Yamaguchi Y., Stallcup W. B. & Tuszynski M. H. NG2 is a major chondroitin sulfate proteoglycan produced after spinal cord injury and is expressed by macrophages and oligodendrocyte progenitors. J. Neurosci. 22, 2792–2803 (2002). 11923444 10.1523/JNEUROSCI.22-07-02792.2002 PMC6758310

[b13] Zhao R. R. *et al.* Lentiviral vectors express chondroitinase ABC in cortical projections and promote sprouting of injured corticospinal axons. J. Neurosci. Methods 201, 228–238 (2011). 21855577 10.1016/j.jneumeth.2011.08.003 PMC3235548

[b14] Ikegami T. *et al.* Chondroitinase ABC combined with neural stem/progenitor cell transplantation enhances graft cell migration and outgrowth of growth-associated protein-43-positive fibers after rat spinal cord injury. Eur. J. Neurosci. 22, 3036–3046 (2005). 10.1111/j.1460-9568.2005.04492.x 16367770

[b15] Massey J. M. *et al.* Increased chondroitin sulfate proteoglycan expression in denervated brainstem targets following spinal cord injury creates a barrier to axonal regeneration overcome by chondroitinase ABC and neurotrophin-3. Exp. Neurol. 209, 426–445 (2008). 17540369 10.1016/j.expneurol.2007.03.029 PMC2270474

[b16] Starkey M. L., Bartus K., Barritt A. W. & Bradbury E. J. Chondroitinase ABC promotes compensatory sprouting of the intact corticospinal tract and recovery of forelimb function following unilateral pyramidotomy in adult mice. Eur. J. Neurosci. 36, 3665–3678 (2012). 23061434 10.1111/ejn.12017 PMC4851235

[b17] Bradbury E. J. & Carter L. M. Manipulating the glial scar: chondroitinase ABC as a therapy for spinal cord injury. Brain Res. Bull. 84, 306–316 (2011). 20620201 10.1016/j.brainresbull.2010.06.015

[b18] Zhao R. R. & Fawcett J. W. Combination treatment with chondroitinase ABC in spinal cord injury-breaking the barrier. Neurosci. Bull. 29, 477–483 (2013). 23839053 10.1007/s12264-013-1359-2 PMC5561941

[b19] Rolls A., Shechter R. & Schwartz M. The bright side of the glial scar in CNS repair. Nat. Rev. Neurosci. 10, 235–241 (2009). 19229242 10.1038/nrn2591

[b20] Okada S. *et al.* Conditional ablation of Stat3 or Socs3 discloses a dual role for reactive astrocytes after spinal cord injury. Nat. Med. 12, 829–834 (2006). 16783372 10.1038/nm1425

[b21] Sato T. *et al.* Differential roles of two *N*-acetylgalactosaminyltransferases, CSGalNAcT-1, and a novel enzyme, CSGalNAcT-2: Initiation and elongation in synthesis of chondroitin sulfate. J. Biol. Chem. 278, 3063–3071 (2003). 12446672 10.1074/jbc.M208886200

[b22] Uyama T., Kitagawa H., Tamura J. & Sugahara K. Molecular cloning and expression of human chondroitin N-acetylgalactosaminyltransferase: the key enzyme for chain initiation and elongation of chondroitin/dermatan sulfate on the protein linkage region tetrasaccharide shared by heparin/heparan sulfate. J. Biol. Chem. 277, 8841–8846 (2002). 11788602 10.1074/jbc.M111434200

[b23] Mizumoto S., Ikegawa S. & Sugahara K. Human genetic disorders caused by mutations in genes encoding biosynthetic enzymes for sulfated glycosaminoglycans. J. Biol. Chem. 288, 10953–10961 (2013). 23457301 10.1074/jbc.R112.437038 PMC3630846

[b24] Nadanaka S. & Kitagawa H. Heparan sulphate biosynthesis and disease. J. Biochem. 144, 7–14 (2008). 18367479 10.1093/jb/mvn040

[b25] Maeda N., Ishii M., Nishimura K. & Kamimura K. Functions of chondroitin sulfate and heparan sulfate in the developing brain. Neurochem. Res. 36, 1228–1240 (2011). 21110089 10.1007/s11064-010-0324-y

[b26] Duchez S. *et al.* Glycotranscriptome study reveals an enzymatic switch modulating glycosaminoglycan synthesis during B-cell development and activation. Eur. J. Immunol. 41, 3632–3644 (2011). 22076801 10.1002/eji.201140865

[b27] Uyama T. *et al.* Molecular cloning and expression of a second chondroitin *N*-acetylgalactosaminyltransferase involved in the initiation and elongation of chondroitin/dermatan sulfate. J. Biol. Chem. 278, 3072–3078 (2003). 12433924 10.1074/jbc.M209446200

[b28] Gulberti S. *et al.* Chondroitin sulfate *N*-acetylgalactosaminyltransferase-1 (CSGalNAcT-1) involved in chondroitin sulfate initiation: Impact of sulfation on activity and specificity. Glycobiology 22, 561–571 (2012). 22156920 10.1093/glycob/cwr172

[b29] Watanabe Y. *et al.* Chondroitin sulfate *N*-acetylgalactosaminyltransferase-1 is required for normal cartilage development. Biochem. J. 432, 47–55 (2010). 20812917 10.1042/BJ20100847 PMC2995422

[b30] Murakami K. *et al.* Nerve injury induces the expression of EXT2, a glycosyltransferase required for heparan sulfate synthesis. Neuroscience 141, 1961–1969 (2006). 16784821 10.1016/j.neuroscience.2006.05.026

[b31] Kantor D. B. *et al.* Semaphorin 5A is a bifunctional axon guidance cue regulated by heparan and chondroitin sulfate proteoglycans. Neuron 44, 961–975 (2004). 15603739 10.1016/j.neuron.2004.12.002

[b32] Ito Z. *et al.* *N*-Acetylglucosamine 6-*O*-sulfotransferase-1-deficient mice show better functional recovery after spinal cord injury. J. Neurosci. 30, 5937–5947 (2010). 20427653 10.1523/JNEUROSCI.2570-09.2010 PMC6632605

[b33] Nozumi M. *et al.* Identification of functional marker proteins in the mammalian growth cone. Proc. Natl Acad. Sci. USA 106, 17211–17216 (2009). 19805073 10.1073/pnas.0904092106 PMC2761339

[b34] Izumikawa T. *et al.* Nematode chondroitin polymerizing factor showing cell-/organ-specific expression is indispensable for chondroitin synthesis and embryonic cell division. J. Biol. Chem. 279, 53755–53761 (2004). 15485872 10.1074/jbc.M409615200

[b35] Wang D. & Fawcett J. The perineuronal net and the control of CNS plasticity. Cell Tissue Res. 349, 147–160 (2012). 22437874 10.1007/s00441-012-1375-y

[b36] Alilain W. J., Horn K. P., Hu H., Dick T. E. & Silver J. Functional regeneration of respiratory pathways after spinal cord injury. Nature 475, 196–200 (2011). 21753849 10.1038/nature10199 PMC3163458

[b37] Massey J. M. *et al.* Chondroitinase ABC digestion of the perineuronal net promotes functional collateral sprouting in the cuneate nucleus after cervical spinal cord injury. J. Neurosci. 26, 4406–4414 (2006). 16624960 10.1523/JNEUROSCI.5467-05.2006 PMC6673998

[b38] Shen Y. *et al.* PTPσ is a receptor for chondroitin sulfate proteoglycan, an inhibitor of neural regeneration. Science 326, 592–595 (2009). 19833921 10.1126/science.1178310 PMC2811318

[b39] Coles C. H. *et al.* Proteoglycan-specific molecular switch for RPTPσ clustering and neuronal extension. Science 332, 484–488 (2011). 21454754 10.1126/science.1200840 PMC3154093

[b40] Bloechlinger S., Karchewski L. A. & Woolf C. J. Dynamic changes in glypican-1 expression in dorsal root ganglion neurons after peripheral and central axonal injury. Eur. J. Neurosci. 19, 1119–1132 (2004). 15016071 10.1111/j.1460-9568.2004.03262.x

[b41] Hsueh Y. P. & Sheng M. Regulated expression and subcellular localization of syndecan heparan sulfate proteoglycans and the syndecan-binding protein CASK/LIN-2 during rat brain development. J. Neurosci. 19, 7415–7425 (1999). 10460248 10.1523/JNEUROSCI.19-17-07415.1999 PMC6782500

[b42] Matsuo I. & Kimura-Yoshida C. Extracellular modulation of Fibroblast growth factor signaling through heparan sulfate proteoglycans in mammalian development. Curr. Opin. Genet. Dev. 23, 399–407 (2013). 23465883 10.1016/j.gde.2013.02.004

[b43] Inatani M. & Yamaguchi Y. Gene expression of EXT1 and EXT2 during mouse brain development. Brain Res. Dev. Brain Res. 141, 129–136 (2003). 10.1016/s0165-3806(03)00010-5 12644256

[b44] Grimpe B. & Silver J. A novel DNA enzyme reduces glycosaminoglycan chains in the glial scar and allows microtransplanted dorsal root ganglia axons to regenerate beyond lesions in the spinal cord. J. Neurosci. 24, 1393–1397 (2004). 14960611 10.1523/JNEUROSCI.4986-03.2004 PMC6730336

[b45] Oudega M. *et al.* Systemic administration of a deoxyribozyme to xylosyltransferase-1 mRNA promotes recovery after a spinal cord contusion injury. Exp. Neurol. 237, 170–179 (2012). 22721770 10.1016/j.expneurol.2012.06.006

[b46] McKillop W. M., Dragan M., Schedl A. & Brown A. Conditional Sox9 ablation reduces chondroitin sulfate proteoglycan levels and improves motor function following spinal cord injury. Glia 61, 164–177 (2013). 23027386 10.1002/glia.22424 PMC4853194

[b47] Tsuji O. *et al.* Therapeutic potential of appropriately evaluated safe-induced pluripotent stem cells for spinal cord injury. Proc. Natl Acad. Sci. USA 107, 12704–12709 (2010). 20615974 10.1073/pnas.0910106107 PMC2906548

[b48] Uetani N. *et al.* Mammalian motoneuron axon targeting requires receptor protein tyrosine phosphatases sigma and delta. J. Neurosci. 26, 5872–5880 (2006). 16738228 10.1523/JNEUROSCI.0386-06.2006 PMC6675220

[b49] Kobayashi S. *et al.* Association of EXT1 and EXT2, hereditary multiple exostoses gene products, in Golgi apparatus. Biochem. Biophys. Res. Commun. 268, 860–867 (2000). 10679296 10.1006/bbrc.2000.2219

[b50] Lairson L. L., Henrissat B., Davies G. J. & Withers S. G. Glycosyltransferases: structures, functions, and mechanisms. Annu. Rev. Biochem. 77, 521–555 (2008). 18518825 10.1146/annurev.biochem.76.061005.092322

[b51] Mu P. *et al.* Systemic delivery of siRNA specific to tumor mediated by atelocollagen: combined therapy using siRNA targeting Bcl-xL and cisplatin against prostate cancer. Int. J. Cancer 125, 2978–2990 (2009). 19422046 10.1002/ijc.24382

[b52] Wang X. *et al.* Ibuprofen enhances recovery from spinal cord injury by limiting tissue loss and stimulating axonal growth. J. Neurotrauma 26, 81–95 (2009). 19125588 10.1089/neu.2007.0464 PMC2913782

[b53] Boato F. *et al.* C3 peptide enhances recovery from spinal cord injury by improved regenerative growth of descending fiber tracts. J. Cell Sci. 123, 1652–1662 (2010). 20406886 10.1242/jcs.066050

[b54] Lin R., Kwok J. C. F., Crespo D. & Fawcett J. W. Chondroitinase ABC has a long-lasting effect on chondroitin sulfate glycosaminoglycan content in the injured rat brain. J. Neurochem. 104, 400–408 (2008). 18005340 10.1111/j.1471-4159.2007.05066.x

[b55] Li H. P. *et al.* Regeneration of nigrostriatal dopaminergic axons by degradation of chondroitin sulfate is accompanied by elimination of the fibrotic scar and glia limitans in the lesion site. J. Neurosci. Res. 85, 536–547 (2007). 17154415 10.1002/jnr.21141

[b56] Yoshioka N. *et al.* Small molecule inhibitor of type I transforming growth factor-β receptor kinase ameliorates the inhibitory milieu in injured brain and promotes regeneration of nigrostriatal dopaminergic axons. J. Neurosci. Res. 89, 381–393 (2011). 21259325 10.1002/jnr.22552

[b57] Kitagawa H., Kinoshita A. & Sugahara K. Microanalysis of glycosaminoglycan-derived disaccharides labeled with the fluorophore 2-aminoacridone by capillary electrophoresis and high-performance liquid chromatography. Anal. Biochem. 232, 114–121 (1995). 8600818 10.1006/abio.1995.9952

