# Supplementary material for: Chondroitin sulphate N-acetylgalactosaminyl-transferase-1 inhibits recovery from neural injury
Source: Nat Commun. 2013 Nov 12;4:2740. doi: 10.1038/ncomms3740 (PMC3831297; doi:10.1038/ncomms3740)
Supplement: Supplementary Figures and Tables — Supplementary Figures S1-S9 and Supplementary Tables S1-S3 [file ncomms3740-s1.pdf]

SUPPLEMENTARY INFORMATION

Chondroitin Sulfate *N*-acetylgalactosaminyltransferase-1 Inhibits Recovery from Neural Injury

Kosei Takeuchi, Nozomu Yoshioka, Susumu Higa Onaga, Yumi Watanabe, Shinji Miyata, Yoshino Wada, Chika Kudo, Masayasu Okada, Kentaro Ohko, Kanako Oda, Toshiya Sato, Minesuke Yokoyama, Natsuki Matsushita, Masaya Nakamura, Hideyuki Okano, Kenji Sakimura, Hitoshi Kawano, Hiroshi Kitagawa & Michihiro Igarashi

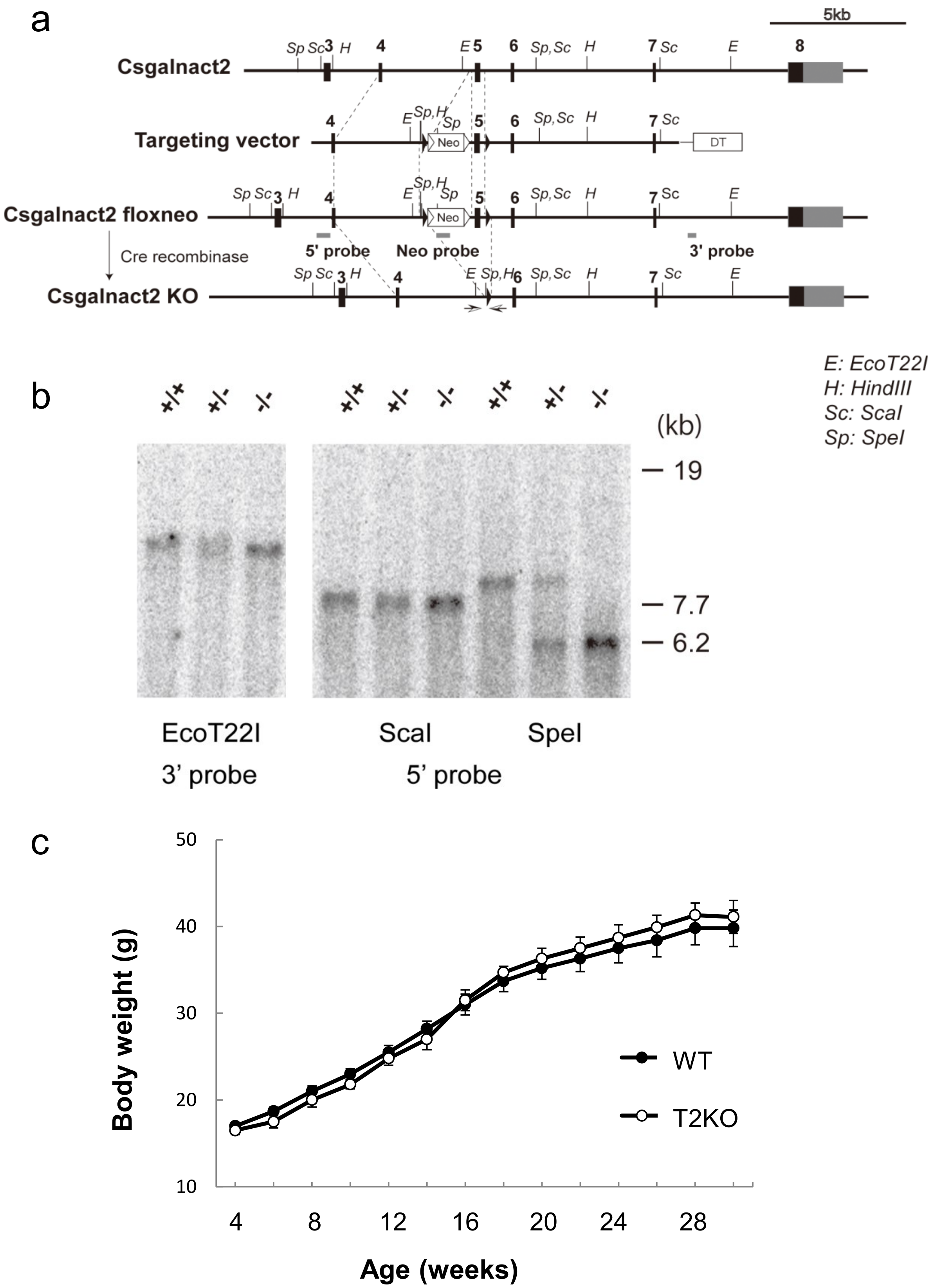

Supplementary Figure. S1 Takeuchi et al.

Supplementary Figure S1. Production of T2KO mice. a. Schematic diagram of the targeting vectors used to produce the T2KO mice. Numbers represent exon numbers (exon 1 is defined as the first exon of this gene and where transcription starts; see also ref. 19). Exon 5 of mouse *T2* encodes a DXD motif thatis essential to GalNAc/Gal transferase activity[31] (see Methods); therefore, we designed the targeting vector with exon 5 located between two loxP sites. b. Southern blot analysis of genomic DNA from T2KO mice confirmed that homologous recombination had occurred. Genomic DNA from littermates was digested with *EcoT*22I for the 3' probe or with *Sca*I or *Spe*I for the 5' probe. The expected genomic fragment size of *T2* alleles were as follows: *EcoT*22I digest and the 3' probe: 10.1 kb (WT) and 9.2 kb (KO); *Sca*I digest and 5' probe: 7.8 kb (WT) and 7.4 kb (KO); *Spe*I digest and the 5' probe: 8.3 kb (WT) and 6.0 kb. **c.** The body weight of wild-type (WT; ●) and T2KO mice (KO; ○), during postnatal development; no significant differences between genotypes were evident (n = 10). Data are expressed as the mean ± SEM.

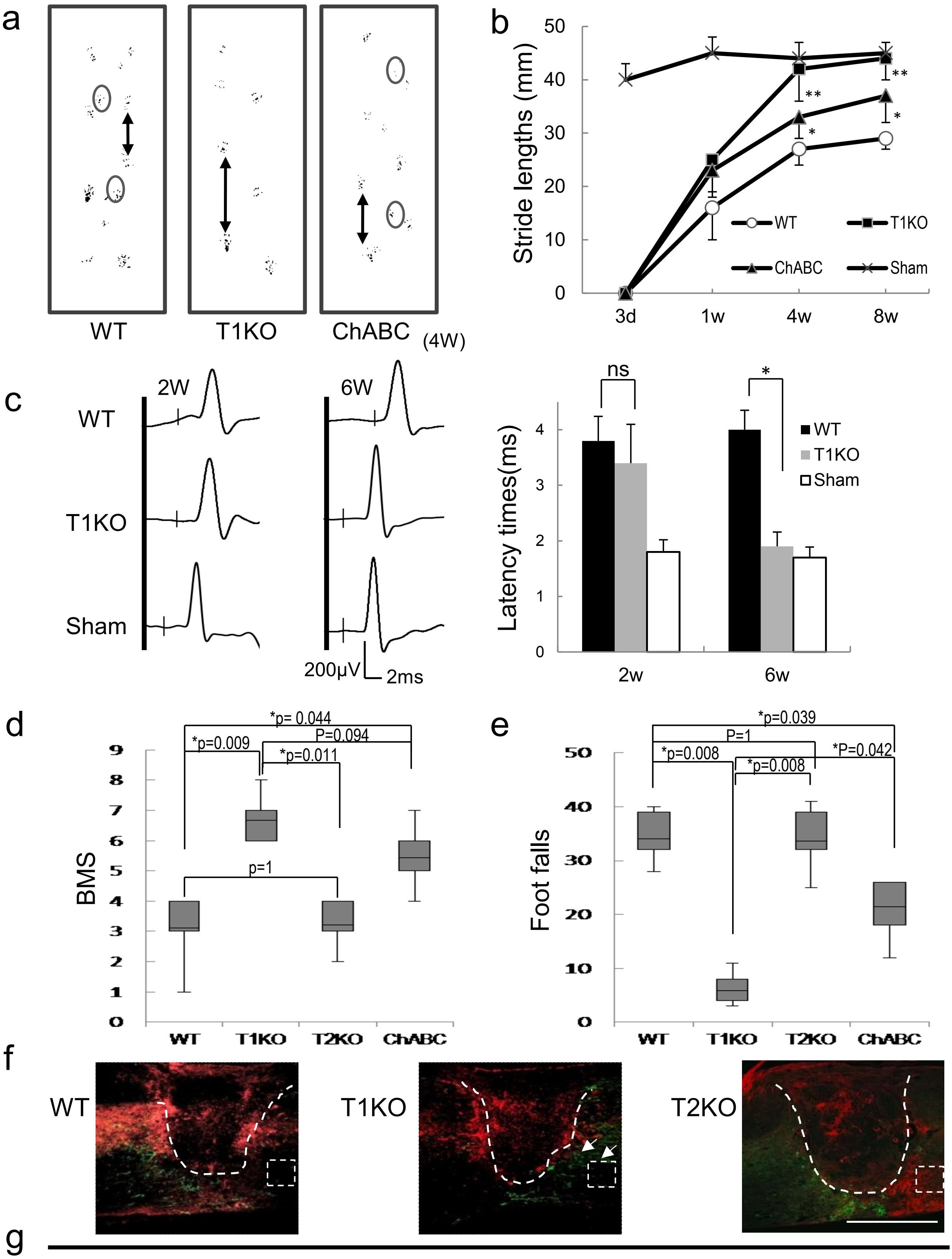

g

	4mm rostral	2mm rostral	Lesion site	2mm caudal	4mm caudal
WT	221 ±32	257 ±21	41 ±21	37±8	50±7
T1KO	301 ±41	398 ±11	97 ±17	392 ±13*	444 ±12*
T2KO	290 ±29	313 ±30	36 ±16	30±7	48±9
ChABC	201 ±69	228 ±47	89 ±26	155 ±20*	298 ±31*

Supplementary Figure. S2 Takeuchi et al.

Supplementary Figure S2. Recovery of the locomotor functions in T1KO mice following SCI. ChABC, ChABC-treated; and Sham, sham-operated mice. (**a)** Representative photographs from footprint tests (4 weeks after SCI). Footprints of WT mice indicate toe dragging and frequent failure of planter placement of the hind paws. In contrast, footprints of T1KO mice illustrate normal plantar placement. Footprints from ChABC mice indicate good stride length, but frequent toe drops (*circles)* and uncoordinated hindpaw placements as well as in *WT*. *Arrows*, the intervals measured. (**b)** Measurement of the interval between footprints. *$p < 0.05$ (Repeated-measures *ANOVA* and the Mann-Whitney *U* test; n=5); T1KO *vs* WT, and T1KO *vs* ChABC. Error bars represents standard deviation for line graphs. **(c)** Recovery of latency for motor-evoked potentials. At 6 weeks after injury, the latency time in T1KO mice is comparable to that in sham-operated mice. Quantitative data are presented as means ± SEM. *$p < 0.05$ (One- way *ANOVA*; n=5). (**d, e**) BMS scoring (**d**) and footfalls (**e**) of WT, T1KO, T2KO, or ChABC-treated (ChABC) mice 8 weeks after SCI; in both tests, the results for T1KO mice were significantly better than those for ChABC mice. Repeated measures *ANOVA* followed by the Bonferroni/Dunn test; *p* values (*$p < 0.05$; n = 9) are shown in the graphs (**d, e**). **(f)** Double immunostaining of 5HT (*green*) and CS-D (*red*). There were 5HT(+) terminals (*arrows*) in the area caudal to the injured site.

Arrows indicate the regrowing or sprouting 5HT(+) terminals in the area caudal to the lesion; such terminals were only evident in T1KO mice. The dotted lines represent the scar borders. Scale bar: 500 μm (*lower*). **(g)** Quantification of the 5HT(+) terminals. The number of 5HT(+) terminals in the ventral horn as seen in coronal sections were counted 6 weeks after injury. Bonferroni's multiple comparison test (Prism 5.04); $*p < 0.05$ (n = 9); T1KO, T2KO, or ChABC *vs* WT. Data are expressed as the mean ± SEM.

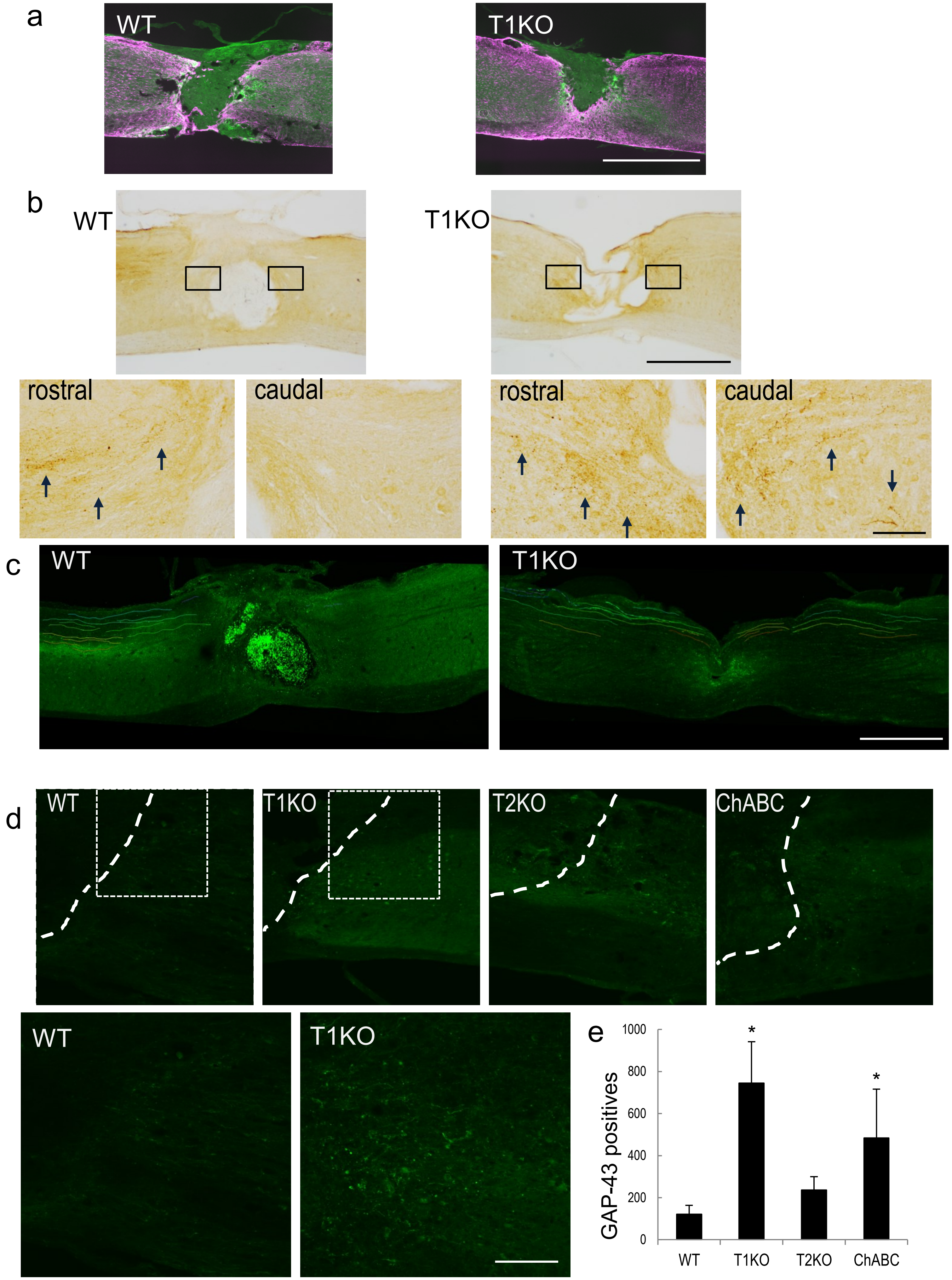

Supplementary Figure. S3 Takeuchi et al.

Supplementary Figure S3. CST axons grow into caudal areas only in T1KO SCI mice. (a) After SCI (2 weeks), T1KO mice had smaller areas that contained glial acidic fibrillary protein (GFAP)-positive cells (*purple*) than did WT mice; this difference indicated that T1KO mice had smaller lesion areas than did WT mice. *Green,* CS-D staining. Scale bar: 1 mm. **(b)** Representative views of BDA-labeled CST fibers. BDA was injected into the motor cortices 8 weeks after SCI. After each injection (2 weeks), the respective mouse was perfused with fixative, and spinal cord sections were prepared and analyzed. (*Upper*) Lower magnification views of sections from WT and T1KO mice. (*Lower*) Higher magnification views of the box areas (both *rostral* and *caudal* areas, respectively) in the *upper* images are shown. The *arrows* indicate axon terminals, and only *T1KO* mice have BDA-positive axon terminals in caudal regions. Scale bar: 1 mm. (**c**) CSTs were reconstructed from images of WT and from T1KO sections. Labeled axons in each image were traced in Imaris software. The traced axons were stacked and superimposed onto an image of BDA staining. Axon segments were reconstructed from the depth of each of the 5 serial sections (200 μm depth). Scale bar: 1 mm. **(d)** Axonal growth paths by GAP-43 staining. (*upper*) The *dotted lines* indicate the margin of the injury sites 2 weeks after SCI in WT, T1KO, T2KO, and ChABC. Scale bar: 500 μm. (*lower*) Higher magnification views of the boxed areas, which include the ventral horns,

in the *upper* images of WT and T1KO sections are shown. Scale bar: 100 μm. The T1KO section has more GAP-43(+) axon terminals, which indicate the growing axons, than does the WT section. (**e**) Quantification of the GAP-43(+) axon terminals. The same regions from 40-μm thick serial sagittal sections from the midline (containing the central canal) were score. Data are expressed as the mean ± SEM. Bonferroni's multiple comparison test; $*p < 0.05$ (n = 5). T1KO, T2KO, or ChABC *vs* WT.

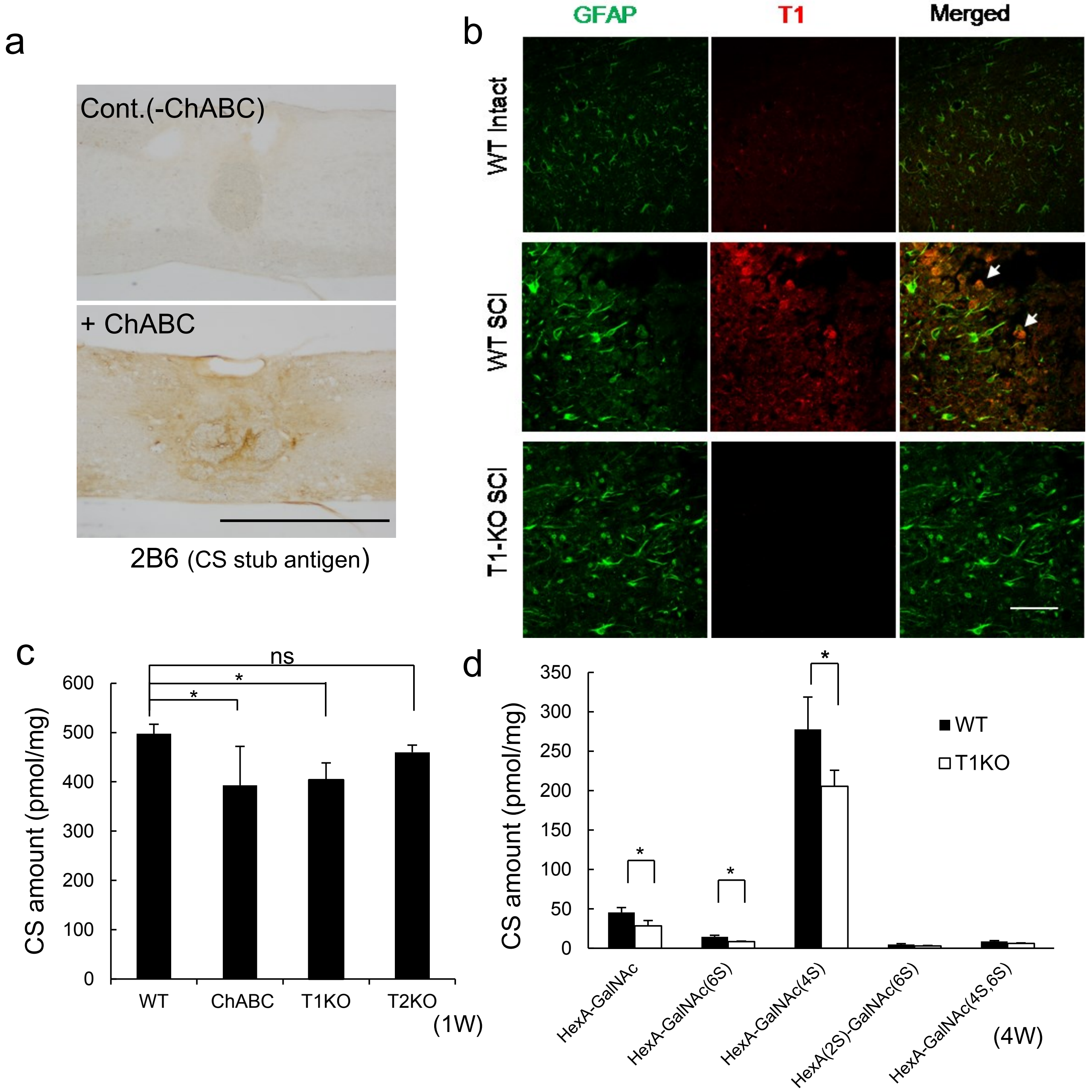

Supplementary Figure. S4 Takeuchi et al.

Supplementary Figure S4. CS distribution and CS-synthesizing enzymes after SCI. **(a)** In injured sites (after 2 weeks of SCI), ChABC treatment broke CS down such that 2B6, an antibody specific to the CS stub, recognized the tissue. Scale bar: 1 mm. **(b)** In WT mice after SCI, T1 (*red*) was upregulated and expressed in some astrocytes that also expressed GFAP (*green*; *arrows*). Scale bar: 50 μm. **(c)** The reduction of CS that was evident in T1KO mice was not evident in T2KO mice. After SCI (1w), the groups were compared with regard to the amount of CS. Data are expressed as the mean ± SEM. $*p < 0.05$ (Bonferroni's multiple comparison test; n = 4). **(d)** Composition of the disaccharides that make up CS in injured spinal cords of WT and T1KO mice. $*p < 0.05$ (Student's *t*-test; n = 5). HexA, hexuronic acid (glucuronic acid or iduronic acid); GalNAc, *N*-acetylglactosamine; S, sulfate. Data are expressed as the mean ± SEM.

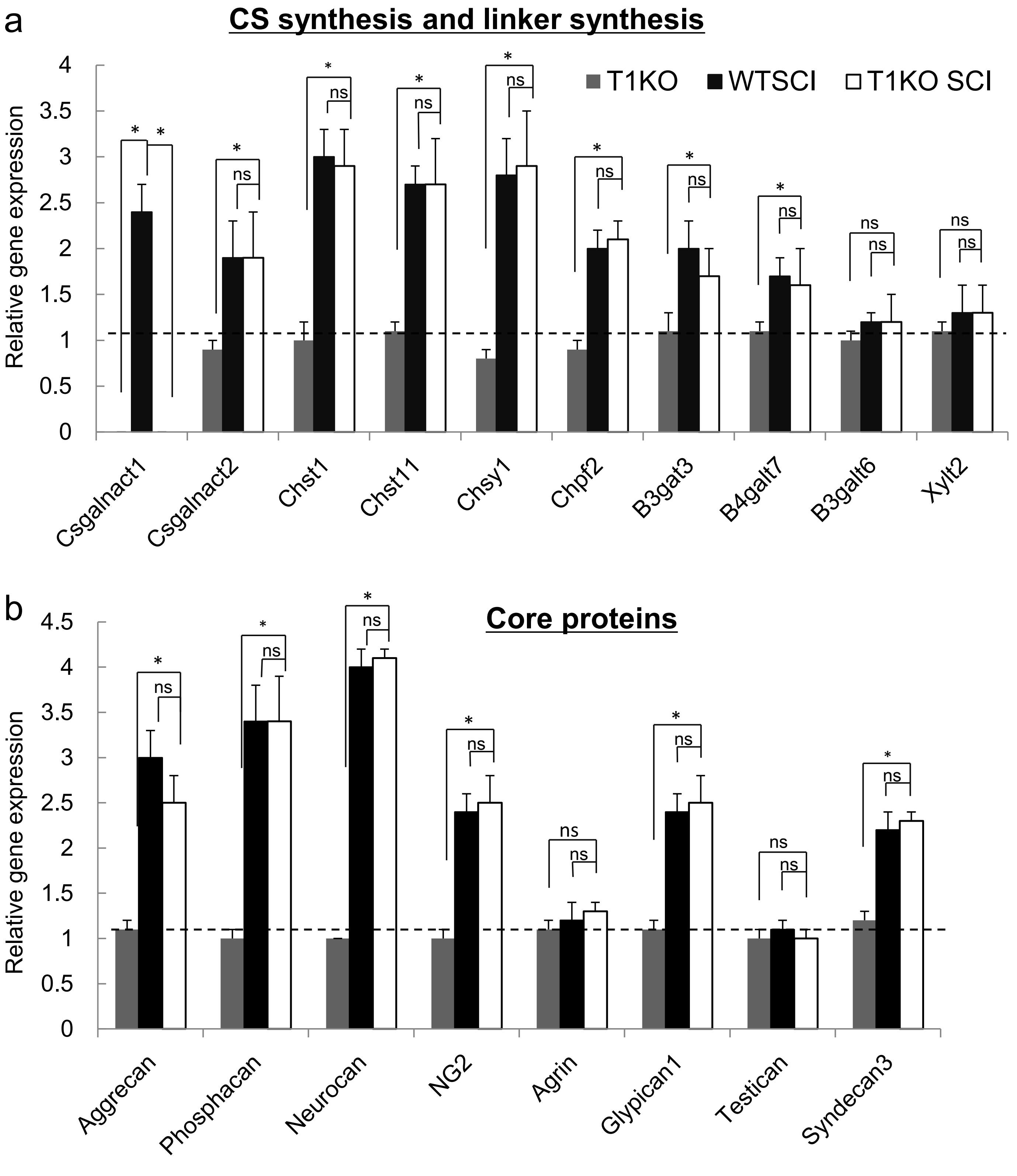

Supplementary Figure. S5 Takeuchi et al.

Supplementary Figure S5. RT-PCR is used to measure expression of mRNAs encoding enzymes necessary for CS synthesis (a) or mRNAs encoding CSPG or HSPG core proteins (b). Neither CS-synthesis enzymes, other than Chst1, nor PG core proteins were upregulated in T1KO mice following SCI. The level of gene expression in WT mice was defined as 1.0 for each gene. Data are expressed as the mean ± SEM. $*p < 0.005$ (Bonferroni's multiple comparison test; n = 15); T1KO SCI *vs* WT SCI, or *vs* T1KO intact.

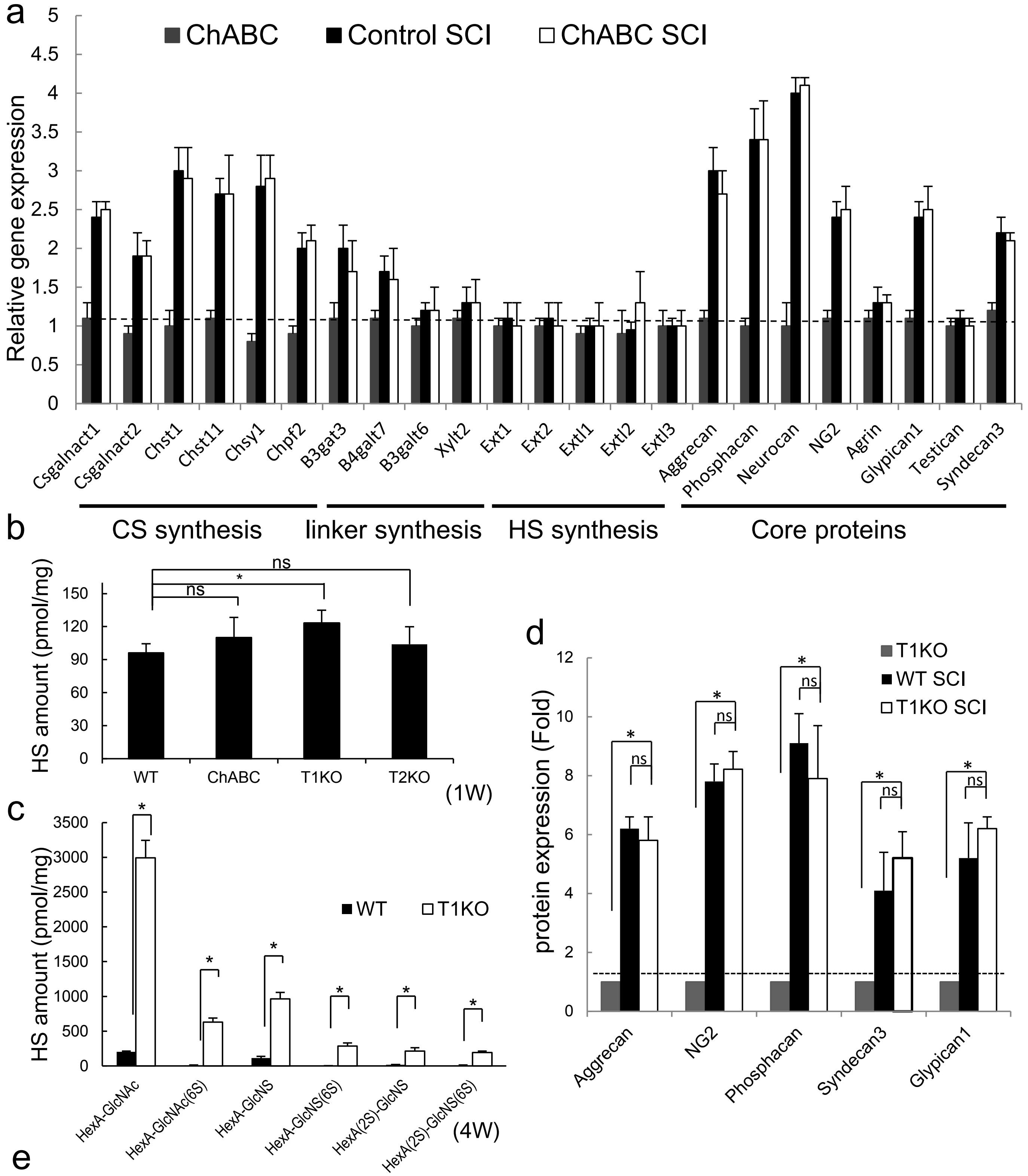

e

	4mm rostral	2mm rostral	Lesion site	2mm caudal	4mm caudal
WT + PBS	201 ±31	221 ±39	54 ±10	80 ±20	71±11
T1KO + PBS	267 ±29	388 ±63	102 ±34*	387 ±61*	301 ±32*
T1KO + HSase	223 ±40	188 ±35	43 ±19	90 ±15	84 ±38
ChABC+ HSase	198 ±33	220 ±41	91 ±40	150 ±20*	289 ±51*

Supplementary Figure. S6 Takeuchi et al.

Supplementary Figure S6. HS expression after SCI. Data are expressed as the mean ± SEM in (a) – (d). (**a)** RT-PCR analysis of the PG-related genes before and after ChABC treatment. ChABC, ChABC treatment after sham operation; Control SCI, SCI in WT mice without ChABC treatment; and ChABC SCI, ChABC treatment after SCI. No significant upregulation of HS-synthesis enzymes was observed (n = 8) in ChABC SCI mice (Bonferroni's multiple comparison test). Also see Fig. 3a and Supplementary Fig. S5. (**b**) Upregulation of HS synthesis was only evident in T1KO mice. The injured sites were analyzed 1 weeks after SCI. *p <0.05 (Bonferroni's multiple comparison test; n = 4). Data are expressed as the mean ± SEM. **(c)** Composition of the disaccharides that make up HS in the injured spinal cords of WT and T1KO mice. *$p < 0.05$ (Student's *t*-test; n = 5). HexA, hexuronic acid (glucuronic acid or iduronic acid); GlcNAc, *N*-acetylglucosamine; GlcN, glucosamine; S, sulfate. **(d)** Quantification of the core protein expression amounts by dot blot analysis (see Fig. 4d). The quantitative data were collected from three mice of each genotype that were each examined 2 weeks after SCI. *$p < 0.05$ (Bonferroni's multiple comparison test; n = 5). **(e)** Analysis of the number of caudal 5HT(+) axon terminals in coronal sections after HSase treatment (after 4 weeks SCI). HSase treatment significantly reduced the number of 5HT(+) terminals in T1KO mice, but not in ChABC-treated mice, indicating that ChABC

treatment did not induce the HS expression but T1KO did (see also **b**). *$p < 0.05$ (Bonferroni's multiple comparison test; n = 9). Data are expressed as the mean ± SEM.

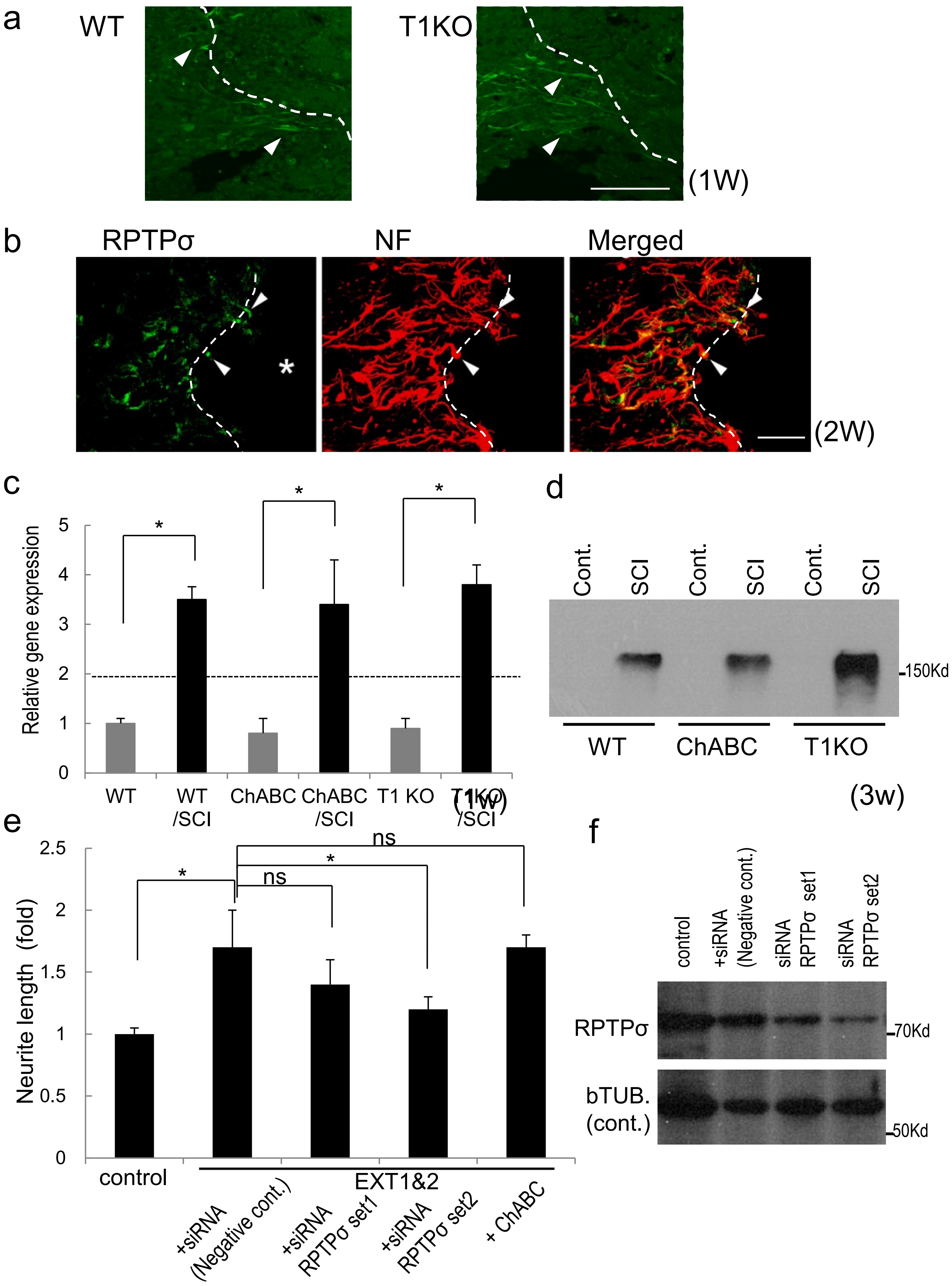

Supplementary Figure. S7 Takeuchi et al.

Supplementary Figure S7. Ext1/Ext2 overexpression in cortical neurons enhances neurite growth in a RPTPσ-dependent manner. (a) Localization of RPTPσ, a receptor for both HS and CS, in regions rostral to the injured site. Scale bar: 20 μm. **(b)** RPTPσ (*green*) co-localized with neurofilaments (NF; *red*) and with some axons (*arrowheads*) beyond the border (*dotted line*) of the glial scar area (*).Scale bar: 20 μm. (**c**) Quantification of *RPTPσ* mRNAexpression by RT-PCR 1 weeks after SCI. The average amount of mRNA in uninjured WT mice was designated as 1.0. Data are expressed as the mean ± SEM; *p < 0. 05 (Student's *t*-test; n = 5). (**d**) Western blots containing proteins from injury site were probed for with anti-RPTPσ antibody. Without SCI, RPTPσ was not expressed. **(e)** Neurite length in cortical neurons that overexpressed RPTPσ. Control, GFP-expressing neurons. Data are expressed as the mean ± SEM; *p < 0.05 (Bonferroni's multiple comparison test; n = 3). (**f)** Immunoblot analysis revealed that the reduction of RPTPσ (72 kDa) was mediated by RNAi of RPTPσ (RNAi set2). βTUB, β-tubulin (55 kDa). Notably, only when siRNA set2 against RPTPσ was introduced, did Ext1 or Ext2 suppression induce increases in neurite length. The molecular masses are shown in the right.

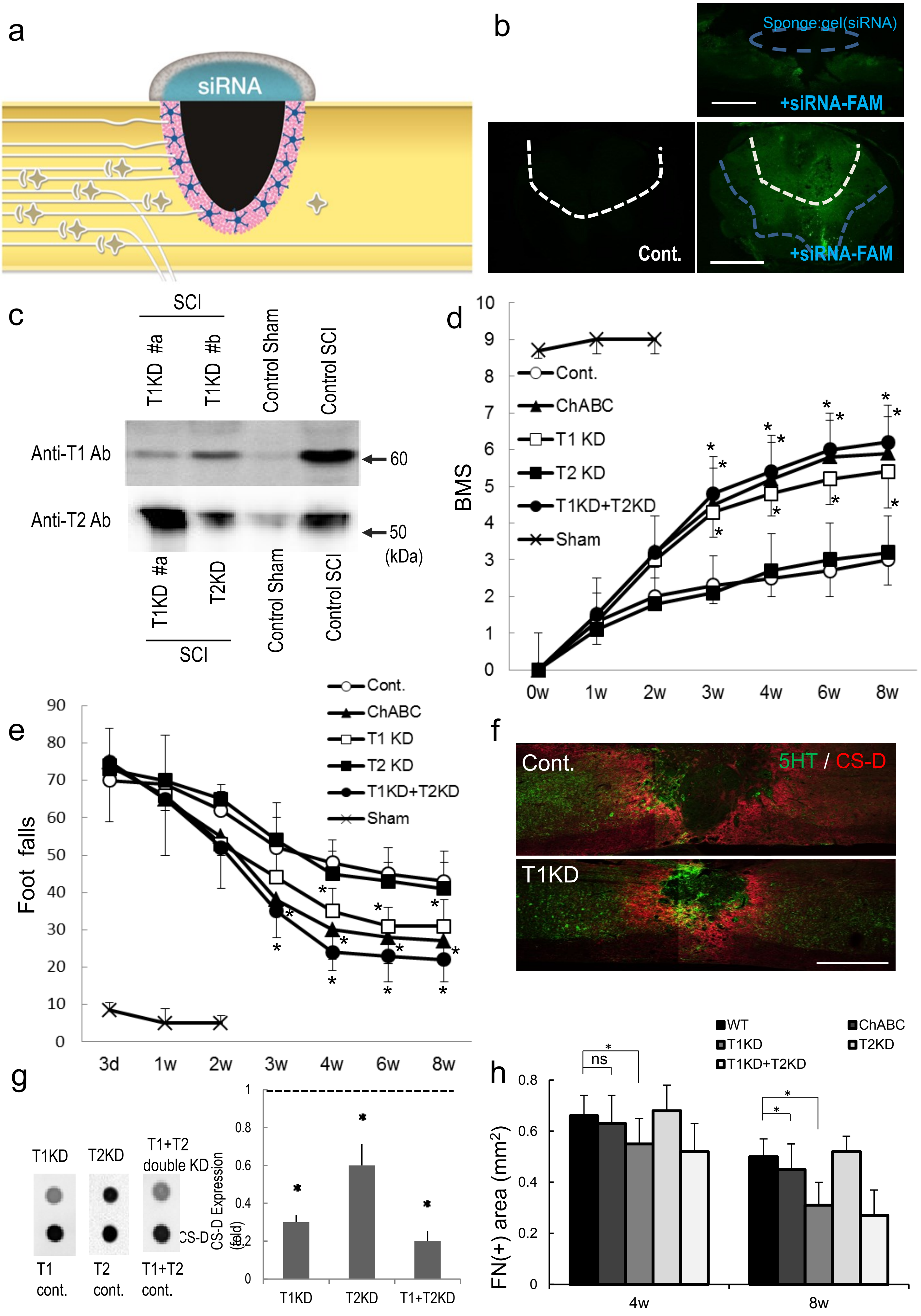

Supplementary Figure. S8 Takeuchi et al.

Supplementary Figure S8. ***In vivo* RNAi targeting T1 is an effective treatment for SCI. (a)** Schematic diagram of *in vivo* application of RNAi via atelocollagen after SCI. Atelocollagen gel (Atelogene®; KOKEN Co. Ltd, Japan) forms a complex with constituent siRNA; a gelfoam patch containing *T1*-targeted siRNA was placed on top of the lesion. The siRNA was gradually delivered to the site of SCI. *Gray*, gelfoam; *blue*, mixture of siRNA and atelocollagen; *black*, fibrotic scar; *pink*, glial scar containing reactive astrocytes. **(b)** siRNA penetrates to a deeper area than the indwelling gelform. Representative micrographs show the region containing fluorescein amide (6-CarboxyFluorescein-Aminohexyl; FAM)-conjugated siRNA. (Upper) The blue dotted line indicates the position where the siRNA and the gel form were placed. (*Lower*) Sagittal section. The *white* and the *blue* dotted lines show margins of the injured area and the regions with siRNAi, respectively. Bar: 1mm. (*Lower left*) Coronal section of the injured site with non-fluorescent siRNA (against *T1*) administration as a control (cont.). (*Lower right*) Administration of the siRNA-FAM (against T1). The FAM-siRNA was administered on day 10 just after the impacted injury. Bar: 500 μm. **(c)** Western blot analysis was conducted with anti-T1 antibody to determine the effects of the siRNA on T1 levels following SCI. SCI-induced elevation of T1 was lower in animals treated with T1-KD#a than in those treated with T1-KD#b. Tissue samples

within a 2-mm range of individual SCI lesion centers were harvested 5 days after SCI; these tissue samples were used to probe western blots. T1-KD#a was used in the subsequent *in vivo* RNAi experiments (**d-f**). **(d, e)** Mice were subjected to SCI and subsequent siRNA-mediated T1-KD or T2-KD; BMS scores (**d**) and footfall tests (**e**) were used to measure recovery from SCI[20]. T1-KD or T2-KD, RNAi-mediated knockdown of T1 (n = 6) or T2 (n = 6), respectively; ChABC, ChABC-treated (n = 6); Sham, Sham-operated (n = 3); Cont, Control (n = 8). Control indicates the administration of a scrambled siRNA derived from T1-KD#a sequences. In (**d)** and (**e**), *post hoc* analyses were conducted using Bonferroni-Dunn for repeated measures *ANOVA*. *$p < 0.05$; KD or ChABC vs. Cont. Error bars represents standard deviation for line graphs. **(f)** Immunohistochemistry was used to detect 5HT(+) axon terminals. 5HT (*green*) and CS (*red*; recognized by CS-D antibody). The upper (Cont) and lower (T1KD) images were taken 4 weeks after SCI. Scale bar: 1 mm. Descending 5HT(+) terminals accumulated in the area distal to (on the right side of the lesion) the site of 4 w after SCI. **(g)** Anti-CS-D antibody was used for dot blot analysis of CS expression after T1-KD, (*Upper*) Dot blot analysis after siRNA. T2-KD, or double KD of both T1 and T2. Although T2-KD#a did specifically inhibit T2 expression (see (**b**)), T2-KD did not inhibit CS expression after SCI (*Csgalnact2* in (**c**)); T1-KD + T2-KD strongly inhibited

the CS synthesis. *: $p < 0.005$ (n = 6; (**a**), (**c**), (**d**)). (*Lower*) The quantitative measurement of the CS-D expression after siRNA (n = 5). The 1.0 value indicates the control (Cont. siRNA treat) expressions (dotted line). In each experiment, each siRNA was administered 2 weeks after SCI. Data are expressed as the mean ± SEM; *$p < 0.05$ (Bonferroni's multiple comparison test; vs 1.0 as the control). **(h)** RNAi-mediated T1-KD caused reduced scars sizes, but not HS upregulation. FN (+; *left*) and GFAP (+; *right*) indicates fibrotic and glial scars, respectively. T1-KD and T1-KD + T2-KD caused significant reductions in scar size, but ChABC treatment did not, and T1-KD alone did not cause sufficient upregulation of HS-synthesis enzymes; taken together these finding suggested that T1-regulated CS synthesis was probably involved in scar sizes, but T1-regulated HS synthesis was not (Supplementary Fig. S9a). Data are expressed as the mean ± SEM; *$p < 0.05$ (n = 5; one -way *ANOVA* and Bonferroni's *post hoc* pairwise comparisons).

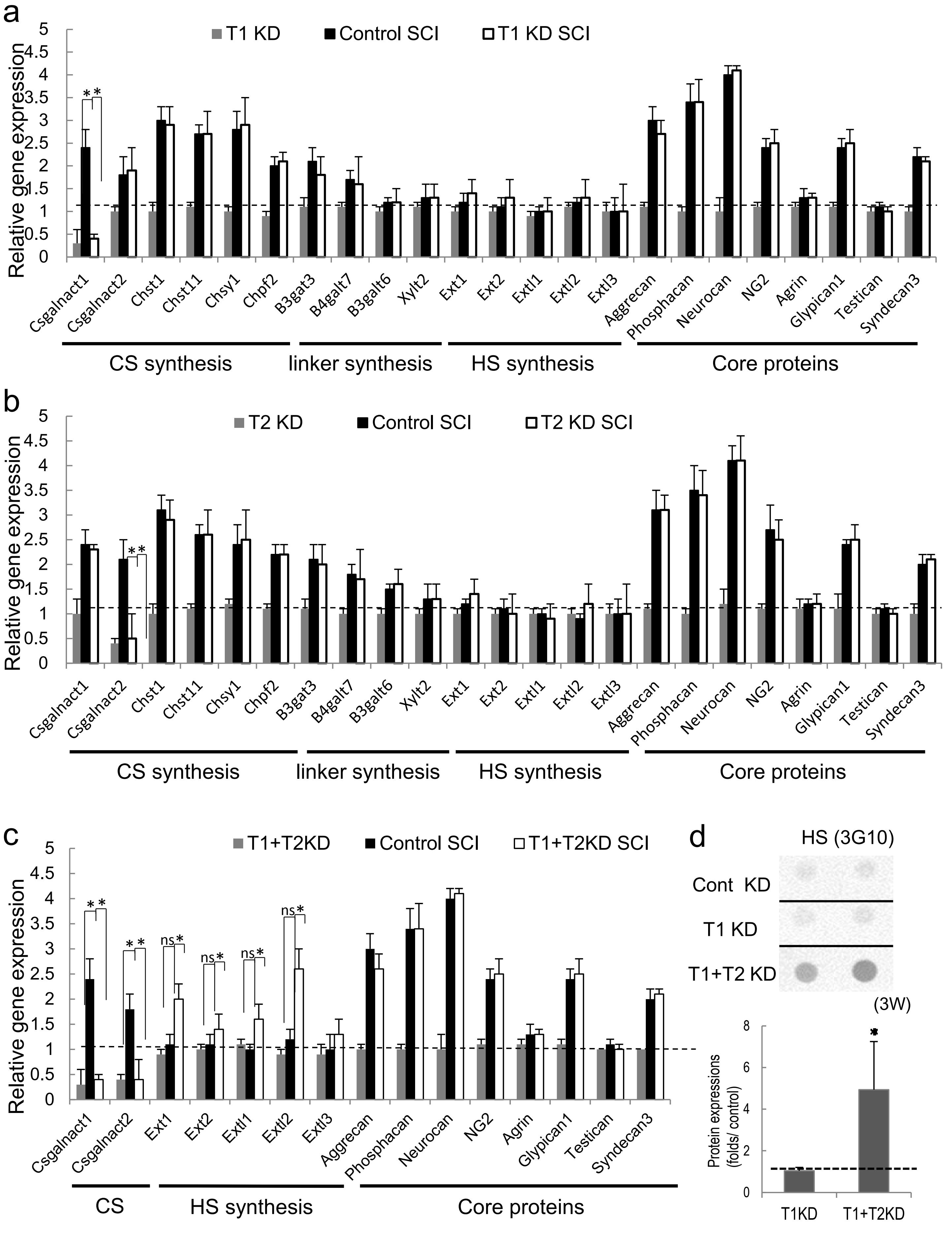

Supplementary Figure. S9 Takeuchi et al.

Supplementary Figure S9. (a, b) Effects of RNAi on the expression of GAG-synthesizing enzymes and the expression of CSPG core proteins following SCI. Data are expressed as the mean ± SEM. Results of the quantitative RT-PCR are shown in (**a**) T1-KD (T1-KD#a in (**c**)), (**b**) T2-KD, and in (**c**) T1-KD + T2-KD (T1 + T2 KD) after SCI. Double knockdown of T1 and T2 (**c**) cause up regulation of HS-synthesis enzymes (Ext1, Ext2, Extl1, and Extl2) as did T1KO (Fig. 3*a*). For each gene, the level of gene expression in control mice was defined as 1.0. *Control* indicates the administration of a scrambled siRNA after SCI with siRNA sequences derived from T1-KD (**a**) or T2-KD (**b**). In (a) and (b), there are no statistically significant differences in these gene expressions between the T1-KD and T2-KD mice (n = 6). *$p < 0.05$ (Bonferroni's multiple comparison test; n = 5; in (**c**)). **(d)** Double KD of T1 and T2 inhibited HS expression 3 weeks after SCI. (*Upper*) A dot blot was probed with the 3G10 antibody to detect HS. Replicate samples are shown. (*Lower*) Quantification of dot-blot signals.* $p < 0.05$ (Bonferroni's multiple comparison test; n = 5).

Supplementary Table S1 Antibodies used in the immunodetection

Antibody	Supplier	Species	Dilution in	
			ABC method	IF, Dot Blot.&Western
Col IV	LSL	Rabbit Polyclonal	1:5000	1:500
CS-D (MO225)	Seikagaku Co.	Mouse Monoclonal IgM	1:200	1:50
CS-A (2H6)	Seikagaku Co.	Mouse Monoclonal IgM	1:200	1:50
CS (CS-56)	Seikagaku Co.	Mouse Monoclonal IgM	-	1:100
FN	Sigma Aldrich	Rabbit Polyclonal	1:2000	1:500
GAP-43	Millipore	Rabbit Polyclonal	1:1000	1:1000
GFAP	Chemicon	Chicken Polyclonal	-	1:2000
GFAP	Daco	Rabbit Polyclonal	1:50	1:50
HS (10E4)	Seikagaku Co.	Mouse Monoclonal IgM	1:200	1:100
HS (HepSS1)	Seikagaku Co.	Mouse Monoclonal IgM	1:200	1:100
HS (3G10)	Seikagaku Co.	Mouse Monoclonal IgG	1:200	1:100
5HT	Sigma Aldrich	Rabbit Polyclonal	1:10000	1:1000
5HT	Immunostar	Rabbit Polyclonal	-	1:1000
NeuN	Chemicon	Mouse Monoclonal	-	1:300
Aggrecan	Abcam	Rabbit Polyclonal	-	1:500
Phosphacan(6B4)	Seikagaku Co.	Mouse Monoclonal IgM	-	1:1000
Syndecan3	R&D	Mouse Monoclonal IgG	-	1:1000
Glypican1	Millipore	Mouse Monoclonal IgG	1:50	1:50
CSGalNacT1	Our group<br>Biochem.J. 432,47-55(2010)	Rabbit Polyclonal	1:1000	1:500
CSGalNacT2	Abnova	Rabbit Polyclonal		1:500
EXT2	Dr. T.Shirasawa<br>BBRC 268,860-867(2000)	Rabbit Polyclonal	1:500	1:100
RPTPσ	R&D	Goat Polyclonal	1:500	
RPTPσ	Dr. M.L.Tremblay<br>J.Neurosci. 26,5872-5880(2006)	Mouse Monoclonal	-	1:1000

Abbreviations used in the Table 1: Col IV (type IV collagen); CS (chondroitin sulfate); FN (fibronectin); GFAP (glial fibrillary acidic protein); TH (tyrosine hydroxylase)

Supplementary Table S2 List of the PCR primers and probes used in RT-PCR analyses

Gene symbol and General preferred protein names	Forward primers(5′-3′)	PrimeTime qPCR probes	Reverse primers(5′-3′)
Csgalnact1(CS-N-acytyl-galactosaminyltransferase1)	GAAAGGGACTGGATGTTGGAG	56-FAM/TCGGAGTTC/ZEN/CTCAACACTTGTAGGC/3IABkFQ	AAATACCTTCTTCCCTGGCTG
Csgalnact2(CS-N-acytyl-galactosaminyltransferase2)	CCATTGTCTATGCCAACCAGG	56-FAM/CAGGTCATC/ZEN/CCAAAGCCAAAGTCTCT/3IABkFQ	TTCCATGTCGAATCCACCG
B3gat3 (Beta-1,3-glucuronyltransferase3)	TGTTTCTTGCCTACTTCCTGG	56-FAM/CCTCTACGC/ZEN/TCTGGTGCAGCTC/3IABkFQ	GATATCCTCAGGTCCTTCTGC
B3galt1 (Beta-1,3-galactosyltransferase1)	GCCTACTTTAAGCTACGTGGT	56-FAM/CTTGGGCTC/ZEN/CAGGTCATTGAGGG/3IABkFQ	TGGCTTCTCTGTTCGTGTATG
B3galt6 (Beta-1,3-galactosyltransferase6)	AGCGATACGTCTTCACTGTG	56-FAM/CGGTGGCTA/ZEN/TGTCCTTTCTGCGG/3IABkFQ	CAACTCTGCGACTACTACCTG
B4galt7 (Beta-1,4-galactosyltransferase7)	TGTCCAAACAGCACTACCAG	56-FAM/TGAATTCTA/ZEN/CCGGCGCATCAAAGGA/3IABkFQ	GTACCCAGTTGTGATTCCCG
B4galt1 (Beta-1,4-galactosyltransferase4)	AGCAACTCGACTATGGCATC	56-FAM/TCGATTGAA/ZEN/CATGGTGTCTCCAGCC/3IABkFQ	AGGTCCACATCACTGAACAC
Chsy1 (Chondroitin sulfate synthase1)	GGAACTTTCTCTTCGTGGGAG	56-FAM/TTGTCTTGG/ZEN/ACCATGTTCTGTAGGCG/3IABkFQ	GATGTATCTGAACCCTCGCTAG
Chst11 (Carbohydrate sulfotransferase 11, C4S-1)	CAAAGTATGTTGCACCCAGTC	56-FAM/AGCAGATGT/ZEN/CCACACCGAAGGG/3IABkFQ	CAGGATGGCAGTGTTGGATAG
Chst1 (Carbohydrate sulfotransferase 1, C6ST)	ATTTCCGGGACCTTGTACAC	56-FAM/CGTCTTGTT/ZEN/CCTGGCATTTGTGGT/3IABkFQ	AATGAGGGATCTGCTTCAGC
Chpf2 (Chondroitin polymerizing factor 2)	CTGAAGACCAGACATAGGGTTG	56-FAM/TTTCTGTTG/ZEN/CTGACCTCGTTGACTCC/3IABkFQ	GCACAGTCTCAAGGATGAGTAG
Xylt2 (Xylosyltransferase 2)	GCCACTGACTATCCAACGAG	56-FAM/TGAACCTGG/ZEN/AATTGTCTCGCCCAT/3IABkFQ	TCACACTCATGGAAAAGCCG
Ext1 (Exostosin-1)	TTGAAGTCTTTACAGGCGGG	56-FAM/ACTGGAGCT/ZEN/GAAAGTGTTGATTGGGA/3IABkFQ	TGAGAGCAGGATGAAATAGCG
Ext2 (Exostosin-2)	TGGGATCGAGGAACAAATCAC	56-FAM/AGCTCCAGG/ZEN/CAACATATTGAACAGCA/3IABkFQ	GTAAGTCCAGGTAGAAAAGCCG
Extl1 (Exostosin-like 1)	TGCCTTTCTCTGAAGTCATCG	56-FAM/CTCGAAGGG/ZEN/AGCATTTCACGGAGG/3IABkFQ	AGTAAGCGGTCCACAGAAAC
Extl2 (Exostosin-like 2)	GGTGTTGATGGTAGACGATGAC	56-FAM/TGAGAAAGC/ZEN/GAAAACAAGGTCCTGGG/3IABkFQ	AGCCTCCGTAACTGTAGATCC
Extl3 (Exostosin-like 3)	TGCCCTGGAATGAGATTGAG	56-FAM/TCTCCACAC/ZEN/CCGAAACCCAAACA/3IABkFQ	GAAACCCACAATGCGATCAC
Hs6st1 (Heparan-sulfate 6-O-sulfotransferase 1)	GGACCGAACTCACCAACTG	56-FAM/TCTCGCAGC/ZEN/AGGGTGATGTAGTAGAA/3IABkFQ	GTCGCCATTCACTCAGGTAG
Hs6st2 (Heparan-sulfate 6-O-sulfotransferase 2)	CTTCAAACTTCAACTCAGGCG	56-FAM/CTTCCCACT/ZEN/CTTCGATGTGCTCCG/3IABkFQ	CCTCCATTCACTCAAGTACCG
Aggrecan (Acan)	AGGTTGCTATGGTGACAAGG	56-FAM/TCGCTGAGG/ZEN/AGATGGAGGGTGA/3IABkFQ	TGGAAGGTGAATTTCTCTGGG
Phosphacan (Ptprz)	GAAAAGGTCTTCAAGGCAAGC	56-FAM/TTGCATCTC/ZEN/CAGTGGGAACTTCTGTC/3IABkFQ	AAAATCTGTCCGCATCAAAGC
Neurocan (Ncan)	TGCCGTAACAATGGGAAGTG	56-FAM/AGGCTTGTG/ZEN/ATGTCGGTGTGGAT/3IABkFQ	GGTGTCTCTTGTGTTTTCTGTG
Agrin (Agrn)	CACCAAAGCCACATAACATTCC	56-FAM/CCACTTCAC/ZEN/AGCAGCGCCTTCTA/3IABkFQ	CTCAACTCCAGCCTTATGCG
Glypican (Gpc1)	TTGTCAGTGATGAGCACCAT	56-FAM/CTGGATGCC/ZEN/GAGTGGAGGAACC/3IABkFQ	CGACTATTGCCGAAATGTGC
NG2 (Cspg4)	CCTTCACGATCACCATCCTTC	56-FAM/ATGACCAAC/ZEN/CCCCTGTTCTCACC/3IABkFQ	AATCATTGTCTGTTCCCCTGAG
Syndecan3 (Sdc3)	GATGATGAACTAGACGACCTCTAC	56-FAM/CTCGAAGTA/ZEN/GCCAGAGCCTGACC/3IABkFQ	CATATCAGGGATGAACCGCAT
Testican (Spock1)	TGAACATCCAACCCAAGGAG	56-FAM/TGACACCAG/ZEN/CATCTTACCCATTTGCA/3IABkFQ	CCAATAGAGTCATCAAGCCTACC
RPTPsigma (Ptprs)	CATCACCTGCCAACCTCTAC	56-FAM/ACGCTTCTC/ZEN/CATCCTGCCCAT/3IABkFQ	TGCATCCATTTCACGTAGGG
Gapdh (glyceraldehyde-3-phosphate dehydrogenase)	AATGGTGAAGGTCGGTGTG	5TexRd-XN/TGCAAATGGCAGCCCTGGTG/3IABkFQ	GTGGAGTCATACTGGAACATGTAG
Actb (actin,beta)	GATTACTGCTCTGGCTCCTAG	5TexRd-XN/CTGGCCTCACTGTCCACCTTCC/3IABkFQ	GACTCATCGTACTCCTGCTTG

*The nomenclatures of gene symbols are followed by the MGI database

Supplementary Table S3 List of the siRNAs used in this study

siRNA target		Target sequence
Csgalnact1 (Chondroitin sulfate-N-acytyl -galactosaminyltransferase1)	Csgalnact1 T1-KD#a	GCAAUCAAAGGCUAUGAAUGA (2179-2199)
	Csgalnact1 T1-KD#b	GGUUCUGUUCAGUCAGUAUAA (1849-1869)
Csgalnact2 (Chondroitin sulfate-N-acytyl -galactosaminyltransferase2)	Csgalnact2 T2-KD#a (T2-KD)	GCAGAGCCAGUAUCUCCUUUG (3411-3431)
	Csgalnact1 T2-KD#b	GGAGGAGAUAGAGAUGCAUCU (1948-1968)
Csgalnact1 control sequence	Csgalnact1 T1-KD#a control	GCUAUCUAUGGCAUUGAUUGA
	Csgalnact1 T1-KD#b control	GGUACUGUACUGUCAGAAUAA
Csgalnact2 control sequence	Csgalnact2 T2-KD#a control (T2-KD control)	GCUGAGCGUGUAACUCCAAUG
	Csgalnact2 T2-KD#b control	GCUGAGCGUGUAACUCCAAUG
RPTPsigma (Ptprs)	RPTPsigma set1	UAAUGACACUCUCCUGUCGUGGUGC (1725-1749 : 25mer)
	RPTPsigma set2	AAUGUUCCCACGUGAAUUGCUGCCC (4209-4233 : 25mer)
RPTPsigma (Ptprs) control seq.	RPTPsigma control	siRNA of scrambled sequence (Invitrogen) was used as a negative control.
Ext1	Ext1 KD	GCAAUCAAAGGCUAUGAAUGA (2179-2200)
Ext1 control seq.	Ext1 control	siRNA of scrambled sequence (Invitrogen) was used as a negative control.
